# Polyphosphazene-Based Nanocarriers for the Release of Camptothecin and Epirubicin

**DOI:** 10.3390/pharmaceutics14010169

**Published:** 2022-01-11

**Authors:** Javier Pérez Quiñones, Cornelia Roschger, Aitziber Iturmendi, Helena Henke, Andreas Zierer, Carlos Peniche-Covas, Oliver Brüggemann

**Affiliations:** 1 Institute of Polymer Chemistry, Johannes Kepler University Linz, Altenberger Straße 69, 4040 Linz, Austria; aitziber.iturmendi@jku.at (A.I.); helena.henke@jku.at (H.H.); oliver.brueggemann@jku.at (O.B.); 2 Department for Cardiac-, Vascular- and Thoracic Surgery, Johannes Kepler University Linz, Kepler University Hospital GmBH, Altenberger Straße 69, 4040 Linz and Krankenhausstraße 7a, 4020 Linz, Austria; cornelia.roschger@jku.at (C.R.); andreas_florian.zierer@jku.at (A.Z.); 3 Facultad de Química, Universidad de La Habana, Zapata S/N entre G y Carlitos Aguirre, La Habana 10400, Cuba; cpeniche2015@yahoo.com

**Keywords:** polyphosphazene, camptothecin, epirubicin, nanocarriers, controlled release

## Abstract

The design and study of efficient polymer-based drug delivery systems for the controlled release of anticancer drugs is one of the pillars of nanomedicine. The fight against metastatic and invasive cancers demands therapeutic candidates with increased and selective toxicity towards malignant cells, long-term activity and reduced side effects. In this sense, polyphosphazene nanocarriers were synthesized for the sustained release of the anticancer drugs camptothecin (CPT) and epirubicin (EPI). Linear poly(dichloro)phosphazene was modified with lipophilic tocopherol or testosterone glycinate, with antioxidant and antitumor activity, and with hydrophilic Jeffamine M1000 to obtain different polyphosphazene nanocarriers. It allowed us to encapsulate the lipophilic CPT and the more hydrophilic EPI. The encapsulation process was carried out via solvent exchange/precipitation, attaining a 9.2–13.6 wt% of CPT and 0.3–2.4 wt% of EPI. CPT-loaded polyphosphazenes formed 140–200 nm aggregates in simulated body physiological conditions (PBS, pH 7.4), resulting in an 80–100-fold increase of CPT solubility. EPI-loaded polyphosphazenes formed 250 nm aggregates in an aqueous medium. CPT and EPI release (PBS, pH 7.4, 37 °C) was monitored for 202 h, being almost linear during the first 8 h. The slow release of testosterone and tocopherol was also sustained for 150 h in PBS (pH 7.4 and 6.0) at 37 °C. The co-delivery of testosterone or tocopherol and the anticancer drugs from the nanocarriers was expected. Cells of the human breast cancer cell line MCF-7 demonstrated good uptake of anticancer-drug-loaded nanocarriers after 6 h. Similarly, MCF-7 spheroids showed good uptake of the anticancer-drug-loaded aggregates after 72 h. Almost all anticancer-drug-loaded polyphosphazenes exhibited similar or superior toxicity against MCF-7 cells and spheroids when compared to raw anticancer drugs. Additionally, cell-cycle arrest in the G2/M phase was increased in response to the drug-loaded nanocarriers. Almost no toxicity of anticancer-drug-loaded aggregates against primary human lung fibroblasts was observed. Furthermore, the aggregates displayed no hemolytic activity, which is in contrast to the parent anticancer drugs. Consequently, synthesized polyphosphazene-based nanocarriers might be potential nanomedicines for chemotherapy.

## 1. Introduction

Cancer is a major cause of death worldwide, with about 9.6 million cancer deaths in 2018 [1,2]. Lung, breast, prostate and colon cancer are the most common and fatal cancers, with 6.1% to 11.6% of newly diagnosed cases and 5.8% to 18.4% mortality [2]. Chemotherapy based on CPT derivatives (i.e., topotecan and irinotecan) and EPI anticancer drugs occasionally fails in the treatment of metastatic or resistant cancers, and patients’ relapse is observed in a moderate-to-low rate. EPI, or, more precisely, epidoxorubicin, is an anthracycline antibiotic capable of inhibiting topoisomerase II, leading to apoptosis of tumor cells due to its interference in the DNA replication process [3]. However, cardiotoxicity related to EPI use is estimated to affect up to 5% of the patients after 15 years of the initial treatment [3]. On the other hand, CPT is the only available topoisomerase I inhibitors widely applied for treatment of several cancers, despite the medium-to-severe side effects of this drug [4,5,6]. This drawback can be circumvented by the use of nanomedicines (i.e., the application of nanotechnology to medicine) [7,8]. The approval of nanoparticles for drug delivery to solid tumors, such as albumin-based paclitaxel nanoparticles has catalyzed the field to develop second-generation nanoparticles for this purpose [9]. In search for nanomedicines for tumor-targeted drug delivery, we worked on CPT and doxorubicin encapsulation in tocopherol-, ergocalciferol- or testosterone-modified cellulose or hyaluronic acid nanogels (2–13 wt% of CPT), with sustained CPT release and good cytotoxic activity against MCF-7 cancer cells [10,11]. DL-α-Tocopherol (vitamin E) and testosterone were chosen as biocompatible substituents of amphiphilic polymers for anticancer drug delivery applications due to their hypocholesterolemic, antioxidant and anticancer effects [12,13,14]. These facts motivated us to synthesize tocopherol and testosterone substituted polyphosphazene nanocarriers for hydrophobic encapsulation of CPT or hydrophilic loading of EPI, and their controlled delivery with strong cytotoxicity against cancer cells and reduced side effects. 

Polyphosphazenes, inorganic–organic hybrid polymers consisting of an inorganic backbone of alternating phosphorus and nitrogen atoms, and organic side groups, introduced during post-polymerization substitution, are of great interest for biomedical applications [15]. Living cationic polymerization yields polymers with controlled molecular weights and narrow polydispersities, while the post-polymerization substitution results in multifunctional polymers, with the polymer characteristics determined by choice of substituents [16]. Their tunable degradation rates and, especially, their degradation to nontoxic degradation products phosphates and ammonia make them highly advantageous for drug delivery applications [3,17]. Polyphosphazenes have been investigated for anticancer drug delivery both in vitro and in vivo, ranging from doxorubicin-loaded polymersomes proving to be less toxic while exhibiting a comparable effectiveness to standard delivery methods, to injectable hydrogels bearing covalently linked CPT showing a better tumor growth inhibition of the intratumorally injected hydrogel compared to the drug being administered alone [18,19]. Macromolecular drugs based on polyphosphazenes bearing covalently linked EPI are claimed to inhibit medullary thyroid carcinoma and small intestinal neuroendocrine tumor cells proliferation coupled with a decrease in cell viability in vitro [20,21]. However, lack of quantitative cytotoxicity data of these polyphosphazenes bearing covalently bonded EPI, as well as missing comparison of their anticancer activity with the observed effect of free EPI, motivated us to keep investigating on synthesis of other polyphosphazenes carrying EPI for antitumor application. Further studies with macromolecular metal prodrugs based on polyphosphazenes bearing Pt(IV) prodrugs showed a 30-fold increase in drug uptake and promising results to overcome drug resistance in vitro with an improvement in loss of tumor volume in vivo [22]. Similarly, ruthenium-bearing macromolecular drugs, providing an increase in solubility of the bound metal drugs, showed an increase in tolerability with a decrease in tumor growth in vivo [23].

In this research, three polyphosphazene-based nanocarriers were synthesized by living cationic polymerization of Cl_3_PNSiMe_3_ to obtain poly(dichloro)phosphazenes, which were further functionalized via post-polymerization substitution with tocopherol or testosterone glycinate and hydrophilic Jeffamine M1000. Synthesized polyphosphazenes were utilized to encapsulate and deliver the anticancer drugs CPT and EPI, respectively. The antiproliferative effect of CPT and EPI on MCF-7 cancer cells was almost unaltered after inclusion in the polyphosphazene-based nanocarriers. Good uptake of CPT- and EPI-loaded polyphosphazene-based aggregates was observed into MCF-7 cancer cells after 6 h. Further studies on MCF-7 3D spheroids also demonstrated the advantages of the synthesized nanocarriers of the anticancer drugs as potential therapeutic agents. To the best of our knowledge, this is the first exhaustive study on CPT- and EPI-loaded polyphosphazene-based nanocarriers aimed for anticancer applications.

## 2. Materials and Methods

### 2.1. Materials

**Figure 1:**
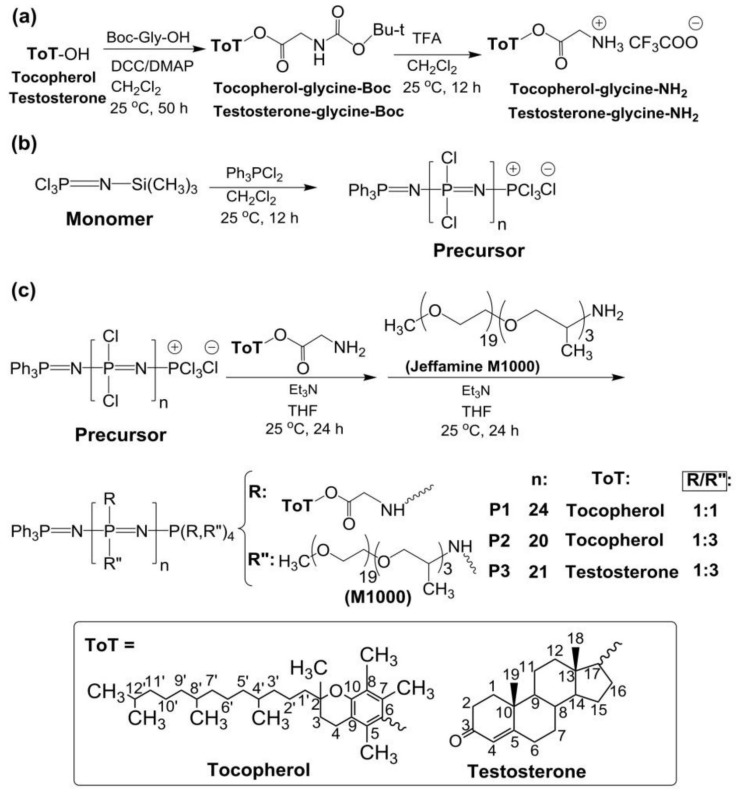

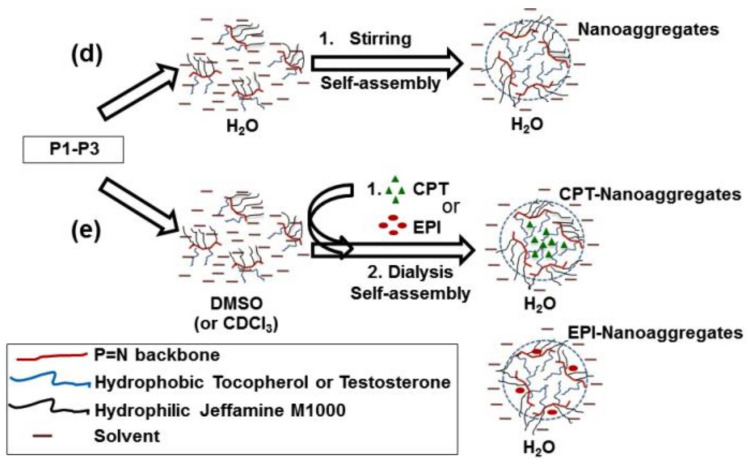
(**a**) Synthesis of tocopherol and testosterone glycinates (ToT-glycine-NH_2_). (**b**) Polymerization of trichlorophosphoranimine to obtain poly(dichloro)phosphazene. (**c**) Post-polymerization functionalization of chlorine atoms with ToT-glycine-NH_2_ and Jeffamine M1000. (**d**) Schematic representation of self-aggregation of polyphosphazenes P1–P3 to form nanoaggregates. (**e**) Encapsulation of CPT and EPI.

Jeffamine M1000 (M_n_ ~1000 Da; Huntsman Performance Products, The Woodlands, TX, USA) is a monoamine terminated polyether formed of ethylene oxide/propylene oxide in a 19/3 ratio. All chemicals and solvents were purchased from Sigma-Aldrich and used as received, without further purification, unless otherwise stated. (*S*)-(+)-Camptothecin (CPT) was purchased from Alfa Aesar (Alfa Aesar GmbH & Co KG, Karlsruhe, Germany). The human breast adenocarcinoma cell line MCF-7 was donated by Professor Dr. Barbara Krammer, University of Salzburg (Salzburg, Austria). Triethylamine was distilled and stored over molecular sieves under argon. All glassware was dried overnight in an oven, at 100 °C, prior to use. Spectra Por 3 cellulose dialysis membranes (Spectrum Laboratories, Inc., Rancho Dominguez, CA, USA) with molecular weight cutoff (MWCO) of 3.5 kDa were used for purification of the synthesized polyphosphazenes. The synthesis and characterization of tocopherol-glycine-Boc, testosterone-glycine-Boc and related deprotected tocopherol-glycine-NH_2_ and testosterone-glycine-NH_2_ are described in the Supplementary Materials (Figure 1a). The monomer *N*-(trimethylsilyl)-trichlorophosphoranimine (Cl_3_P = N–Si(CH_3_)_3_) was synthesized according to the literature procedure [20,24].

### 2.2. Synthesis of the Polymers

The synthesis of the poly(dichloro)phosphazene precursor was conducted via living cationic polymerization of monomer trichlorophosphoranimine (Cl_3_P = N–Si(CH_3_)_3_) with triphenylphosphine dichloride ((C_6_H_5_)_3_PCl_2_) (Figure 1b) [25,26]. The synthesis of polymer P1 is briefly described in the Supplementary Materials. For polymers P2 and P3, the ratio of monomer to initiator was maintained, and the ratio of substituent tocopherol or testosterone to Jeffamine M1000 was adjusted differently (Table 1). These syntheses were performed in a glovebox (MBRAUN, Inc., Stratham, NH, USA), under argon. The structures of the polymers P1–P3 and the synthetic route followed in this work are shown in Figure 1.

Polyphosphazenes P1–P3 formed nanoaggregates in aqueous medium when stirred overnight at 1 mg/mL in water or phosphate buffer saline solution (PBS, pH 7.4) (Figure 1d).

### 2.3. CPT and EPI Loading in Polyphosphazene Nanocarriers

CPT or EPI was incorporated into the polyphosphazene-based nanocarriers, using a solvent exchange/precipitation method, with lyophilization [11]. To this end, approximately 10 mg of P1, P2 or P3 and 1.2–1.5 mg of CPT or EPI dissolved in 10 mL of DMSO were stirred overnight at room temperature in darkness. The formation of CPT- or EPI-loaded polyphosphazene nanoaggregates and removal of CPT and EPI excess was carried out with dialysis against distilled water (2 L, 1 time, 5 h). CPT-loaded polyphosphazenes prepared with 1.5 mg of CPT and 10 mg of P1 (1.5CPT-P1), approximately 1.0–1.2 mg of CPT and 10 mg of P1–P3 (CPT-P1, CPT-P2 and CPT-P3) (slightly brown waxy solids), and EPI-loaded polyphosphazenes prepared with 1 mg of EPI and 10 mg of P1 or P2 (EPI-P1 and EPI-P2) (red waxy solids) were obtained after lyophilization.

### 2.4. Anticancer Drug Content and Drug Release Studies

The content of CPT in CPT-loaded polyphosphazene nanocarriers (Supplementary Materials Figure S1) and the in vitro release of CPT during drug release studies were determined by HPLC measurements with diode array detection (DAD), based on calibration curves of CPT in PBS (pH 7.4) at 254 and 370 nm (Supplementary Materials Figures S2 and S3) (Rt 4.5 min, R^2^ 0.999 and detection limits 0.41–12.2 µg/mL) [27,28]. Similarly, the content of EPI in EPI-loaded polyphosphazene nanocarriers (Supplementary Materials Figure S4) and in vitro EPI released were determined by HPLC with DAD detection at 254 nm and 475 nm [20,29], using calibration curves of epirubicin hydrochloride in PBS (pH 7.4) (Supplementary Materials Figures S5 and S6) (Rt 6.1 min, R^2^ ranged from 0.985 to 0.986 and detection limits from 0.41 to 12.2 µg/mL).

In vitro release studies of CPT and EPI, respectively, were performed in PBS (pH 7.4) at 37 °C. To this end, 2.0 mL of CPT- or EPI-loaded polyphosphazenes 1.5CPT-P1, CPT-P1, CPT-P2, CPT-P3, EPI-P1 and EPI-P2 (2.5 mg/mL) in PBS at pH 7.4 was placed in dialysis cups (MWCO 3.5 kDa, Slide–A–Lyzer Mini Dialysis Devices, ThermoScientific, Rockford, IL, USA) and immersed in 10 mL of the release medium stirred at 100 rpm. The entire release medium was replaced at every required time point, and it was analyzed by using HPLC detection at 254 and 370 nm for CPT releases, and 254 and 475 nm for EPI releases [20,27,28,29]. Tocopherol or testosterone delivery of carrier polyphosphazene P1–P3 in PBS (pH 7.4 and pH 6.0) at 37 °C was evaluated with UV spectrophotometry, using the calibration curves of tocopherol or testosterone in PBS at pH 7.4 and 6.0 (testosterone $\varepsilon_{249}^{\mathrm{PBS}}$ = 17,594 M^−^^1^ cm^−1^, testosterone $\varepsilon_{249}^{\mathrm{PBS}}$ = 16,613 M^−1^ cm^−1^, tocopherol $\varepsilon_{290}^{\mathrm{PBS}}$ = 8960 M^−1^ cm^−1^, tocopherol $\varepsilon_{290}^{\mathrm{PBS}}$ = 10,768 M^−1^ cm^−1^) (Supplementary Materials Figures S7–S10). Release experiments were conducted in triplicate. All drug-release data were fitted to a SWeibull2 model (cumulative release (%) = a − (a − b) * exp(−(k * time(hours))^d^)). The CPT and EPI release data were also adjusted to the Korsmeyer–Peppas model (linear fitting of log(cumulative release (%)) *=* k * log(time (hours) + m).

### 2.5. Characterization

FTIR spectra of the polymers and precursors were measured with a PerkinElmer Spectrum 100 FTIR spectrophotometer (PerkinElmer Ltd., Buckinghamshire, UK), equipped with an attenuated reflectance (ATR) accessory, 4 cm^−1^ resolution.

UV–Vis spectra were acquired on a Perkin Elmer Lambda 25 UV/VIS spectrophotometer.

ESI–MS spectra of the tocopherol-glycine-NH_2_ and testosterone glycine-NH_2_ were acquired by using an Agilent Technologies mass spectrometer LC/MSD TrapSL (Agilent Technologies, Vienna, Austria) equipped with electrospray ionization (ESI) interface operated in positive mode. The samples were injected dissolved in chloroform or methanol.

Proton, attached proton test (APT) carbon and phosphorus NMR spectra of the samples in CDCl_3_ were determined in a Bruker Avance III 300 spectrometer (Bruker, Switzerland). The NMR data was processed with TopSpin 3.6.1 (Bruker BioSpin GmbH, Rheinstetten, Germany).

Molecular-weight estimations of the polyphosphazenes P1–P3 were carried out in a Viscotek GPCmax gel permeation chromatograph (GPC) with a PFG column (PSS, Mainz, Germany) (300 mm × 8 mm and 5 µm particle size). A Viscotek TDA 305 Triple Detector Array (Malvern, Kassel, Germany) was used for refractive index, viscometer and light scattering detections of the eluates. A total of 100 µL of the polymers was eluted with 10 mM LiBr in *N*,*N*-Dimethylformamide at a flow rate of 1 mL/min at 60 °C. Molecular weights were estimated by using conventional calibration against linear polystyrene standards (PSS, Mainz, Germany). The GPC measurements were acquired and processed with OmniSEC 5.12.467 (Malvern, Kassel, Germany).

Dynamic light scattering (DLS) studies were performed by using a Malvern Zetasizer Nano ZS (Malvern Instruments Ltd., Malvern, UK), with standard acquisition settings for backscatter detection at 173°. The aggregates dispersed in Milli-Q water or PBS (1 mg/mL), were filtered through a 0.45 µm nylon filter and hydrodynamic sizes (*d*_h_) were measured in a DTS1070 polystyrene cell at room temperature.

The thermal behavior of the polymers was studied by using differential scanning calorimetry (DSC) and thermogravimetric analysis (TGA). A total of 5 mg of the polymers in closed aluminum pans was cooled and heated from −80 to 400 °C, using a DSC Q2000 (TA Instrument, Eschborn, Germany). A 10 °C/min ramp and 20 mL/min of nitrogen flow was used for all DSC determinations. TGA of the samples (~5 mg) placed on platinum pans were registered by using a TGA Q5000 instrument (TA Instruments, Eschborn, Germany). Samples were heated from 40 to 900 °C, with a 10 °C/min ramp and a nitrogen flow 25 mL/min for TGA measurements.

TEM images were registered with a JEM-2011 FasTEM (Jeol Ltd., Tokyo, Japan). A total of 10 µL of aggregates in water (1 mg/mL) was deposited on Pioloform coated cooper grids (Plano GmbH, Wetzlar, Germany). Negative staining with 1% uranyl acetate solution was applied to the samples.

Atomic Force Microscopy (AFM) images (10 × 10 µm and 2 × 2 µm) were taken with MFP 3D-Stand Alone AFM (Asylum Research, Oxford, UK) with the cantilever OMCL-AC160TSA of Olympus (Olympus Europa SE & Co. KG, Hamburg, Germany), at a resonant frequency of 300 kHz and spring constant of 26 N/m, 50–70% set point and scan rate of 1 Hz. An 80 µL droplet of a 1 mg/mL aqueous dispersion of the polymers was deposited on a silicon wafer spin coated at 40 Hz for 6 s.

Scanning Electron Microscopy (SEM) micrographs were recorded with a field emission Zeiss Gemini 1540 XB SEM (Zeiss, Jena, Germany) operated at 7–8 kV, using a secondary electron detector. A drop of polymer dispersions in deionized water (1 mg/mL) was deposited on a silicon wafer (0.35 × 0.35 cm^2^), the water was evaporated overnight and samples were gold coated with a HUMMER X (Anatech Ltd., Alexandria, VA, USA) sputter-coater before measurements.

HPLC determinations were performed with a 1290 Infinity UPLC system (Agilent Technologies, Vienna, Austria), with a Zorbax Eclipse Plus C18 column (2.1 × 50 mm and 1.8 µm particle size) thermostated at 30 °C and DAD. A total of 20 µL of the samples was eluted at 0.5 mL/min with a 25% ACN in water (*v*/*v*) containing 0.1% formic acid (*v*/*v*) in isocratic mode. UV detection was carried out at 254, 370 and 475 nm, with a bandwidth of 4 nm, using 540 nm as the wavelength reference [20]. The HPLC measurements were programmed and processed with ChemStation for LC 3D systems (Rev. B.04.03-SP2, Agilent Technologies, Vienna, Austria).

### 2.6. Cell Culture, Cell Uptake and Cytotoxicity Tests

Human breast adenocarcinoma cell line MCF-7 and primary human lung fibroblasts were seeded in Dulbecco’s modified Eagle’s medium (DMEM)–high glucose. The medium was complemented with 10% fetal bovine serum, 1% penicillin (all Sigma-Aldrich, St. Louis, MO, USA) and 1% L-glutamine (PAA laboratories, Leonding, Austria). The cell line MCF-7 and the primary lung fibroblast were kindly provided by Professor Barbara Kramer, Paris-Lodron-University, Salzburg, Austria. Then 70–80% confluent cells were studied, with seeding densities of 1 × 10^4^ cells/well (96-well plates), 3 × 10^4^ cells/well (8-well glass bottom µ-Slides), 1 × 10^5^ cells/well (12-well plates) and 5 × 10^5^ cells/well (6-well plates). 

For the cell uptake experiments, MCF-7 cells were grown overnight in 8-well glass-bottom µ-Slides (ibidiTreat, Ibidi, Graefeling, Germany). The day after, the medium was changed to serum-free medium (control) or CPT-loaded polyphosphazene nanoaggregates dispersions at 0.1 mg/mL and incubated for 4 h. Afterwards, 50 nM of LysoTracker Yellow HCK-123 (Invitrogen, Waltham, USA) was added, and incubation was continued for another 2 h. For the EPI-loaded polyphosphazene nanoaggregate dispersions, cells were incubated for 6 h at a concentration of 0.1 mg/mL and subsequently counterstained with 1 µg/mL Hoechst 33,342 (Fluka, Buchs, Switzerland). Fluorescence imaging was performed by using an Olympus IX73 inverted microscope with DAPI channel for the 1.5CPT-P1, CPT-P1, CPT-P2 and CPT-P3 nanoaggregates (λ_excitation_ = 345 nm, λ_emission_ = 455 nm) and FITC channel for the LysoTracker Yellow HCK-123 (λ_excitation_ = 494 nm, λ_emission_ = 518 nm). Similarly, EPI-P1 and EPI-P2 nanoaggregates (λ_excitation_ = 550 nm, λ_emission_ = 565 nm) were analyzed by using CY3 channel, while DAPI channel was used for the cell nuclei marker Hoechst 33342 (λ_excitation_ = 345 nm, λ_emission_ = 455 nm).

To determine the cytotoxic effects of the blank and CPT- or EPI-loaded polyphosphazene-based nanocarriers, primary human lung fibroblasts and MCF-7 cells were grown overnight in 96-well plates. The day after, cells were treated with various concentrations of unloaded polymers and CPT- or EPI-loaded polyphosphazene nanoaggregate dispersions in serum free medium. After 48 h, the medium was changed to complete growth medium, and 50 μL of XTT (2,3-bis(2-methoxy-4-nitro-5-sulfophenyl)-2*H*-tetrazolium-5-carboxanilide, Sigma-Aldrich, St. Louis, MA, USA) reagent was added to each well. After 3 h of incubation, absorbance was determined at 490 nm with a GloMax^®^ Multimode Microplate Reader (Promega, Madison, WI, USA). Data were processed by using OriginPro 2015 (OriginLab, Northampton, MA, USA).

### 2.7. Annexin V/PI Assay

Cells in complete growth medium were seeded into 12-well culture plates. The day after, the medium was changed to serum-free medium with compounds at various concentrations. After 48 h, supernatants were transferred into Eppendorf tubes. Cells were detached with 500 µL Accutase^®^ solution (Sigma Aldrich, St. Louis, MO, USA) and added to the respective supernatant. The suspension was centrifuged for 5 min at 1500 rpm. After removal of the supernatant, the cell pellet was washed twice with 1 mL DPBS and re-suspended in 98.5 µL of 1X Annexin V Buffer with 1 µL Annexin V-APC (Immuno Tools, Friesoythe, Germany) and 0.5 µL of 1 mg/mL propidium iodide (PI; Sigma Aldrich, St. Louis, MO, USA). After 15 min of incubation at room temperature in the dark, 200 µL of 1X Annexin V Buffer was added, and samples were measured on a CytoFLEX flow cytometer (Beckman Coulter, Brea, CA, USA). Analyses from at least three independent experiments were performed with the Kaluza 1.5a software (Beckman Coulter, Brea, CA, USA).

### 2.8. Cell Cycle Analysis

Cells grown overnight in 6-well plates were treated with compounds at various concentrations for 72 h. Afterwards, the supernatants were harvested and transferred into Eppendorf tubes. Detachment of cells was performed with 500 μL Accutase^®^ solution. After centrifugation at 1500 rpm for 5 min, the pellet was washed with 1 mL DPBS. The supernatant was removed, and the cell pellet was re-suspended in 100 μL DPBS. For the fixation of cells, 1 mL of ice-cold 70% ethanol was added slowly to the cells, under constant agitation. After freezing for at least 1 h at −20 °C, the cell suspension was washed twice with 2 mL DPBS. The obtained pellet was stained for 15 min with 5 μL PI (0.4 mg/mL), 5 μL RNase solution (1 mg/mL, Sigma Aldrich, St. Louis, MO, USA) and 90 μL DPBS. Subsequently, cells were measured on a CytoFLEX flow cytometer (Beckman Coulter, Brea, CA, USA). At least three independent experiments were conducted and analyzed with the Kaluza 1.5a software (Beckman Coulter, Brea, CA, USA).

### 2.9. Spheroid Generation

Cells were seeded in a BIOFLOAT FLEX (faCellitate, Mannheim, Germany) coated non-TC treated U-bottom 96-well plate. Subsequently, the plate was centrifuged for 5 min at 300× *g* to facilitate spheroid formation. 

### 2.10. Compound Uptake into Spheroids 

After an incubation period of 4 days, the spheroids were treated with 0.1 mg/mL of the indicated compounds for 72 h. The spheroids were washed twice with DPBS and CPT-loaded aggregates were stained with PI for 10 min to visualize dead cells. EPI-loaded aggregates were counterstained with Hoechst 33,342 (Fluka, Buchs, Switzerland). Fluorescence imaging was performed by using an Olympus IX73 inverted microscope with DAPI channel for Hoechst 33,342 and the CPT-loaded aggregates (λ_excitation_ = 345 nm, λ_emission_ = 455 nm) and Cy3 channel for PI and the EPI-loaded aggregates (λ_excitation_ = 550 nm, λ_emission_ = 565 nm).

### 2.11. Cell Viability Analysis of Spheroids

After an incubation period of 4 days, the spheroids were exposed to increasing concentrations of the compounds for 72 h. Cell viability was determined with the CellTiter-Glo*^®^* 3D Cell Viability Assay (Promega, Madison, WI, USA), as described in the manufacturer’s protocol. Luminescence was measured with a GloMax^®^ plate reader (Promega, Madison, WI, USA).

### 2.12. Hemolysis Assay

A total of 4 mL of whole blood was obtained from a healthy human volunteer (GFBV13 application form, Red cross Blood Center, Linz, Austria), directly drawn into Vacuette^®^ EDTAtubes (GreinerBio-One, Kremsmünster, Austria) to prevent coagulation. The blood was centrifuged for 5 min at 2500× *g* to obtain a red blood cell (RBC) pellet. After discarding the plasma, the RBCs were washed twice with 150 mM NaCl (Merck, Darmstadt, Germany) and three times with PBS (pH 7.4). Afterwards, the RBC was diluted 1:50 with PBS at pH 6.2 or pH 7.4, and 200 μL of RBC suspension was exposed to the nanoparticles for 24 h at 37 °C. Suspension with 1% Triton-X (Merck, Darmstadt, Germany) or PBS pH 7.4 served as positive and negative control, respectively. After centrifugation for 5 min at 500× *g*, 100 μL of the supernatant was transferred into a flat-bottom 96-well plate. To avoid interference of EPI carrying samples with strong absorbance at 450 and 490 nm, absorbance was measured with a GloMax^®^ microplate reader (Promega, Madison, WI, USA) at 405 nm. Percent of hemolysis was calculated as follows: $\%\;\mathrm{hemolysis}\;=\left[\frac{\left(\mathrm{Absorbance}\;\mathrm{sample}\;-\mathrm{Absorbance}\;\mathrm{negative}\;\mathrm{control}\right)}{\mathrm{Absorbance}\;\mathrm{positive}\;\mathrm{control}}\right]\times 100$.

### 2.13. Statistical Analyses

All data were normalized and are reported as average value ± standard deviation. Data were analyzed by using a one-way ANOVA with Tukey’s post-test for multiple comparison (Statgraphics Plus 5.1, Professional Edition). Group of means with no significant differences are presented with the same letter (*p* > 0.05). Means with significant differences are presented with different letters or the same letter but different numbers following the letter (*p* < 0.05). 

## 3. Results and Discussion

### 3.1. Synthesis and Characterization of Polyphosphazenes P1–P3

First, linear poly(dichloro)phosphazenes ([NPCl_2_]_n_) with a polymerization degree (DP) close to 25 were synthesized via living cationic polymerization of trichlorophosphoranimine initiated with (C_6_H_5_)_3_PCl_2_ in CH_2_Cl_2_ at room temperature (Figure 1b). Tocopherol-glycine-NH_2_ or testosterone-glycine-NH_2_, respectively (Figure 1a), and Jeffamine M1000 (PEO-PPO-NH_2_) were subsequently introduced via post-polymerization functionalization of the [NPCl_2_]_n_, first introducing the bulky tocopheryl or testosterone moieties and later the linear PEO-PPO-NH_2_ substituent (Figure 1c). Complete chlorine substitution of [NPCl_2_]_n_—which is necessary to avoid later uncontrolled polyphosphazene degradation via hydrolysis, due to highly labile P-Cl bonds—was ensured by using an excess of Jeffamine M1000 [25], and was confirmed by ^31^P{^1^H} NMR spectroscopy measurements with the disappearance of characteristic peaks of unsubstituted chlorine atoms of RClP = N (Supplementary Materials Figure S18) [30]. The amphipathic polyphosphazenes formed with hydrophobic tocopherol/testosterone and hydrophilic Jeffamine moieties might adopt a micellar conformation in water (Figure 1d) [31,32,33,34]. The micelle-like structure allows the hydrophobic encapsulation of lipophilic drugs in the core or the loading of hydrophilic drugs on the shell (Figure 1e).

### 3.2. Structural Characterization and Morphologies

**Figure 2:**
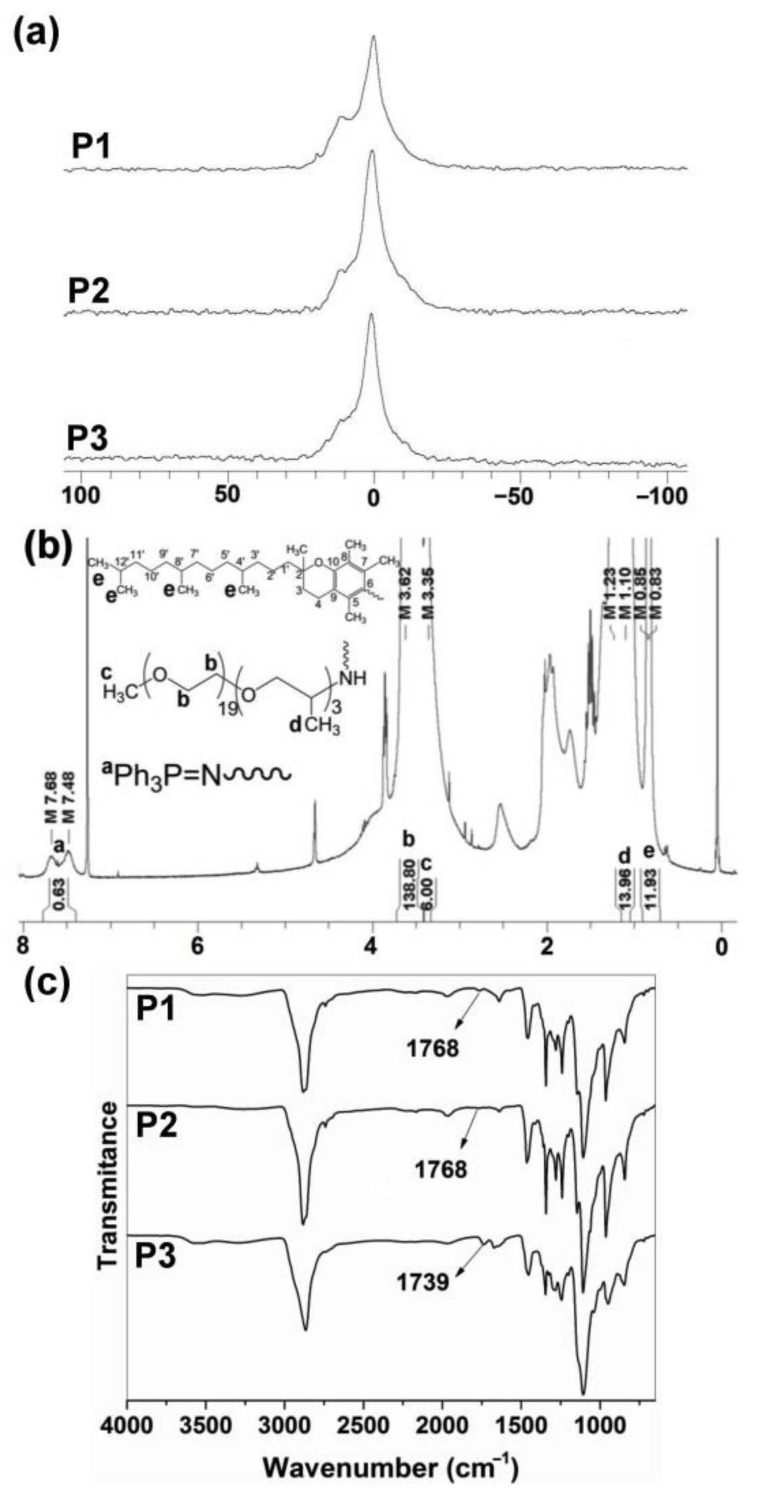
(**a**) ^31^P{^1^H} NMR spectra of P1–P3 polyphosphazenes in CDCl_3_. (**b**) ^1^H NMR spectrum of P1 in CDCl_3_. DP was calculated as $DP=\frac{15}{I_{Ph_{3}P-}}=\frac{15}{Ia}$. (**c**) FTIR spectra of P1–P3 polyphosphazenes (see structures in Figure 1).

^31^P{^1^H} NMR spectra of polymers P1–P3 showed only broad peaks at 0.0 to 1.0 ppm (Figure 2a), as is characteristic of polyphosphazene backbone with Jeffamine M1000 used as substituent [20,26]. The hydrophilic Jeffamine M1000 substituent in the synthesized polymers P1–P3 makes them suitable to be dispersed in aqueous solutions, allowing for their later application as carriers for drug delivery of CPT and EPI. The integration of aromatic protons (C_6_*H*_5_)_3_P = N – (7.60 ppm) of P1–P3 was approximately 15/(20 to 24) when referred to integration of –O–C*H*_2_–C*H*_2_–O– (3.62–3.63 ppm) and C*H*_3_O– (3.36 ppm) protons of Jeffamine M1000, with a degree of polymerization (DP) near to 25 (Table 1) (Figure 2b; Supplementary Materials Figures S21 and S22) [20]. GPC measurements (Supplementary Materials Figures S24–S26) reproduced the trend of calculated number average molecular weights of the polymers (${\bar{M}}_{n}^{P2}>{\bar{M}}_{n}^{P3}>{\bar{M}}_{n}^{P1}$) and showed low polydispersities (M_w_/M_n_ from 1.24 to 1.48), consistent with previous reports [20,25,26].

ATR–FTIR spectroscopy also demonstrated the post-polymerization functionalization of the [NPCl_2_]_n_ with the tocopherol and testosterone glycinate (Figure 2c). Thus, C=O absorption peaks of tocopherol and testosterone glycinate were observed in the polymers P1–P3 at 1768 and 1739 cm^−1^ respectively. Additionally, C=O absorption peak of ketone due to testosterone substituent was observed at 1672 cm^−1^.

The average hydrodynamic diameters (*d*_h_) and the sizes of dried nanoaggregates (*d*_AFM_), as an indication of the self-assembly process, seem to be influenced by the hydrophobicity of the synthesized polyphosphazenes (Table 2). P1, the most hydrophobic synthesized polyphosphazene with tocopherol to Jeffamine M1000 ratio of 1:1, showed the smaller hydrodynamic sizes in water and PBS (~22 nm) and size of dried nanoaggregates (~27 nm) among the three polymers. Polyphosphazenes P2 and P3, with a tocopherol or testosterone to Jeffamine M1000 ratio of 1:3, formed aqueous nanoaggregates of 96–134 nm which upon drying resulted in 42–58 nm nanoparticles (Table 2). However, the inclusion of a hydrophobic drug (CPT) or a hydrophilic drug (EPI) during the self-assembly process via solvent exchange from DMSO to water significantly impacts the hydrodynamic sizes of the aggregates formed by the polyphosphazenes P1–P3 (Figure 1e and Table 2). In this sense, the CPT loading in P1–P3 polymers resulted in a hydrodynamic sizes increase from 22 nm (P1) to 142 nm for CPT-P1 and 198 nm for 1.5CPT-P1, from 127 nm (P2) to 158 nm for CPT-P2 and from 96 nm (P3) to 194 nm for CPT-P3. The most hydrophobic polymer P1 was capable to load the maximal CPT quantity (CPT-P1 with 10.7 wt% CPT content) when compared with CPT-P2 and CPT-P3, all prepared using the same drug loading conditions (1.2 mg of CPT and 10 mg of each polymer (P1–P3)). This might be due to the 50 mol% content of tocopherol in P1, capable to stablish π–π interactions with the aromatic backbone of CPT in the core of the P1 aggregates. However, all CPT bearing polymers dispersed at 1 mg/mL in PBS (pH 7.4) are capable to carry 80–100-fold times the CPT soluble in water as the parent drug (1.3 mg/L) [35]. Interestingly, EPI loading in the outer hydrophilic shell formed by Jeffamine M1000 chains of the P1 and P2 aggregates (Figure 1d) was significantly less efficient than CPT encapsulation, attaining 0.3–2.4 wt% EPI content, with an encapsulation efficiency of 3–23%. It resulted in bigger hydrodynamic sizes of approximately 250 nm in EPI-P1 and EPI-P2. 

**Figure 3:**
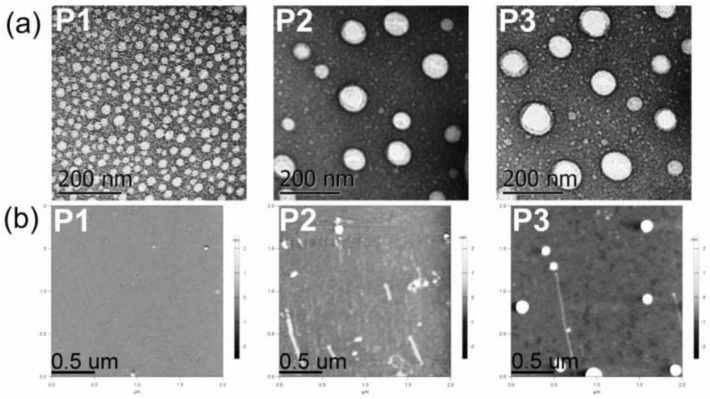
(**a**) TEM micrographs of P1–P3 dried nanoaggregates at 21,000× magnification. (**b**) AFM micrographs of P1–P3 dried nanoaggregates (see structures in Figure 1).

Electron microscopy techniques (TEM and SEM) and AFM allowed us to gain an insight into the morphology and particulate structure of dried P1–P3 polyphosphazene-based nanocarriers. TEM technique depicted the dried P1–P3 nanoaggregates as 20−100 nm rounded particles (Figure 3a). AFM also showed the formation of small rounded nanoaggregates with average sizes of approximately 20 to 60 nm for polymers P1–P3 (Table 2 and Figure 3b). Similarly, SEM displayed small nanoparticles of 20–50 nm and a few 100–250 nm aggregates for polymers P1 and P2 (Supplementary Materials Figure S33). The significant shrinkage of P2 and P3 aggregates upon drying (~60%) might be due to water loss and collapse of the soft micelle-like structure.

Therefore, soft CPT-loaded polyphosphazene-based aggregates (*d*_h_ 142–198 nm) and EPI-loaded polyphosphazene-based aggregates (*d*_h_ ~250 nm) have the appropriate sizes (100–200 nm), deformability and number average molecular weight (<30 kDa) to allow an extended serum circulation with reduced renal clearance, which allow accumulation of these anticancer-drug-loaded nanocarriers in the cancer tissues via passive diffusion and aided by the enhanced permeability and retention (EPR) effect associated to the leaky vasculature and poor lymphatic drainage of tumor tissues [36,37]. Once these CPT- and EPI-loaded nanocarriers deliver their cargo to the cancer cells, the polyphosphazene-based nanocarriers are expected to degrade into innocuous phosphates and ammonia at physiological conditions over time, avoiding harmful long-term accumulation in the body [17].

### 3.3. Drug Delivery Studies

**Figure 4:**
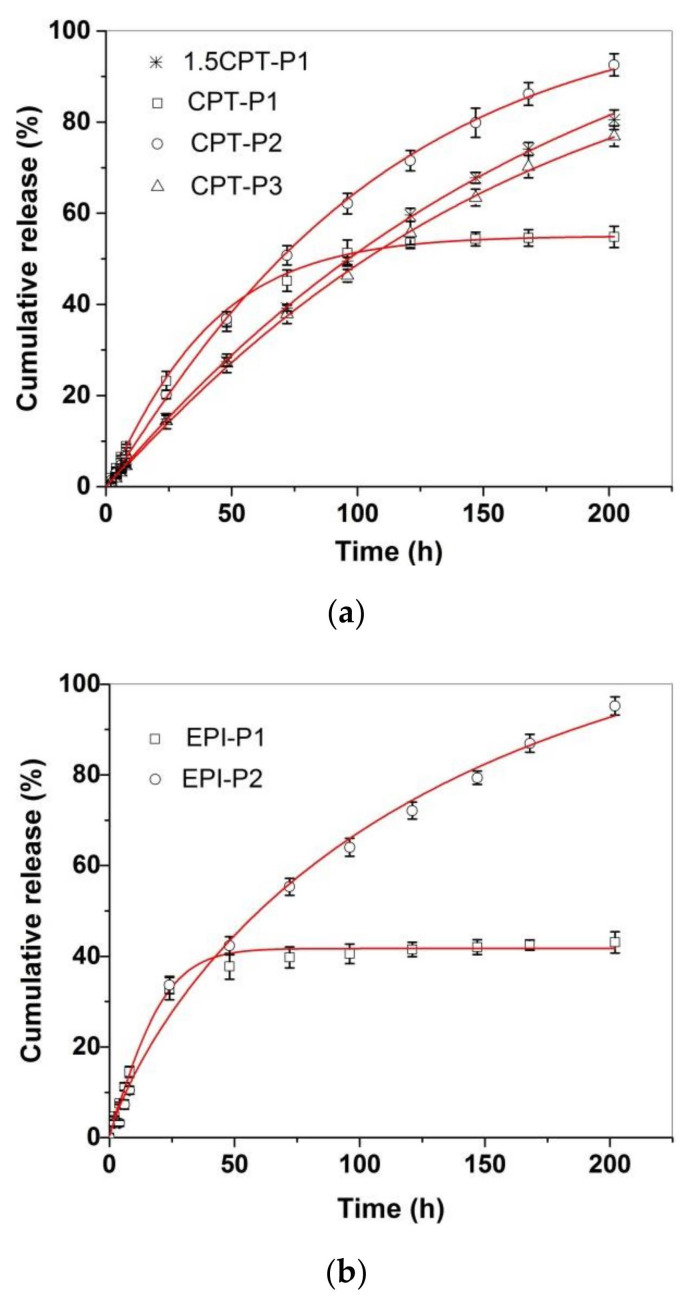
In vitro release profiles of (**a**) CPT-loaded aggregates and (**b**) EPI-loaded aggregates in PBS (pH 7.4) at 37 °C, adjusted to a SWeibull2 function.

Figure 4 shows the in vitro CPT and EPI release profiles of CPT- and EPI-loaded aggregates in simulated physiological conditions (PBS at pH 7.4, 37 °C). All CPT and EPI releases appeared almost linear for the first 8 h, with adjusted R^2^ over 0.97 and slope ranging from 0.54 to 1.09%/h for CPT and 1.22 or 1.86%/h for EPI (Supplementary Materials Table S1 and Figures S34 and S35). CPT release reached approximately 93% of encapsulated CPT for CPT-P2, 81% for 1.5CPT-P1, 77% for CPT-P3 and approximately 55% for CPT-P1 after 8 days. Then, CPT-P1 with the smaller 142 nm aggregates in PBS and second higher CPT content released a lower quantity of CPT. The EPI release showed to be quite different for 250–253 nm aggregates EPI-P1 and EPI-P2. EPI-P2 aggregates with only 0.3 wt% content of EPI released quantitatively (~95%) on the eight day the loaded EPI, while the EPI-P1 aggregates with 2.4 wt% content of EPI reached a 43% of the drug released on the eighth day (Figure 4). Therefore, releases appeared to be controlled by the hydrodynamic size of the aggregates in PBS and the drug content. The CPT and EPI release profiles were adjusted to a Weibull model that describes the drug release from a matrix [38,39].

In this sense, the drug-release data fit the SWeibull2 function well, with adjusted R^2^ over 0.99 and d values of ~1 for all drug-loaded aggregates (except for EPI-P1), associated to an exponential drug release behavior (Figure 4 and Supplementary Materials Table S2) [38]. The anticancer-drug-release data were also adjusted to the Korsmeyer–Peppas model to better clarify the molecular interactions and factors leading to the anticancer drug release (Supplementary Materials Table S3 and Figures S36 and S37) [38,39]. The slope of the linear fitting to the Korsmeyer–Peppas model for CPT releases of CPT-loaded aggregates ranged from 0.85 to 1.03, which is associated with an anomalous diffusion mechanism of release [39]. Similarly, EPI-P1 and EPI-P2 aggregates released EPI with a slope of 0.79 and 0.95 respectively, associated with an anomalous diffusion mechanism of release (slope > 0.5) characteristic of weak interactions between the anticancer drugs and the polymers [39]. Finally, degradation of polyphosphazenes P1–P3 in PBS (pH 7.4 and 6.0) at 37 °C via release of tocopherol and testosterone was also investigated (Supplementary Materials Figures S38–S40). The hydrolysis of the ester bond in the tocopherol glycinate moiety of the polyphosphazenes P1 and P2 resulted in the release of 5.5% and 10% of tocopherol incorporated in P1 after 1 day and 6 days, respectively, in PBS (pH 7.4) at 37 °C. In similar conditions, 7% and 15% of tocopherol incorporated in P2 was released after 1 day and 6 days, respectively. In the same way, hydrolysis of the testosterone glycinate fragment of the polyphosphazene P3 in simulated physiological conditions (PBS, pH 7.4) at 37 °C, resulted in the release of approximately 6% and 21% of the testosterone after 1 day and 6 days, respectively. Slightly acidic medium (PBS, pH 6.0) significantly catalyzed the hydrolysis of P1–P3 polymers. Tocopherol released increased until 10% and 16% at 1 day and 6 days, respectively, for P1. It increased until 15% and 24% at 1 day and 6 days for P2. A 15% and 29% of testosterone was released at day 1 and day 6, respectively, for P3. Therefore, the anticancer drug-loaded polyphosphazenes P1–P3 should deliver both the CPT (1.5CPT-P1, CPT-P1, CPT-P2 and CPT-P3) or EPI (EPI-P1 and EPI-P2) and the testosterone (P3-based nanocarriers) or tocopherol (P1- and P2-based nanocarriers) when administered to the patients, due to simultaneous slow diffusion of the anticancer drugs from the nanocarriers and hydrolysis of glycinate linker with release of testosterone or tocopherol. Interestingly, the degradation of P1–P3 and the CPT and EPI releases in almost neutral conditions (PBS pH 7.4) might progress slowly enough during the first 8 h to allow the accumulation of the aggregates in the slightly more acidic cancer tissues and the cancer cell uptake via passive diffusion and EPR. That is why in vitro cell uptake and cytotoxicity studies were carried out in a first approach to assess the potential application of the synthesized polyphosphazene-based nanocarriers for chemotherapy.

### 3.4. Cell Uptake and Cytotoxic Activity

**Figure 5:**
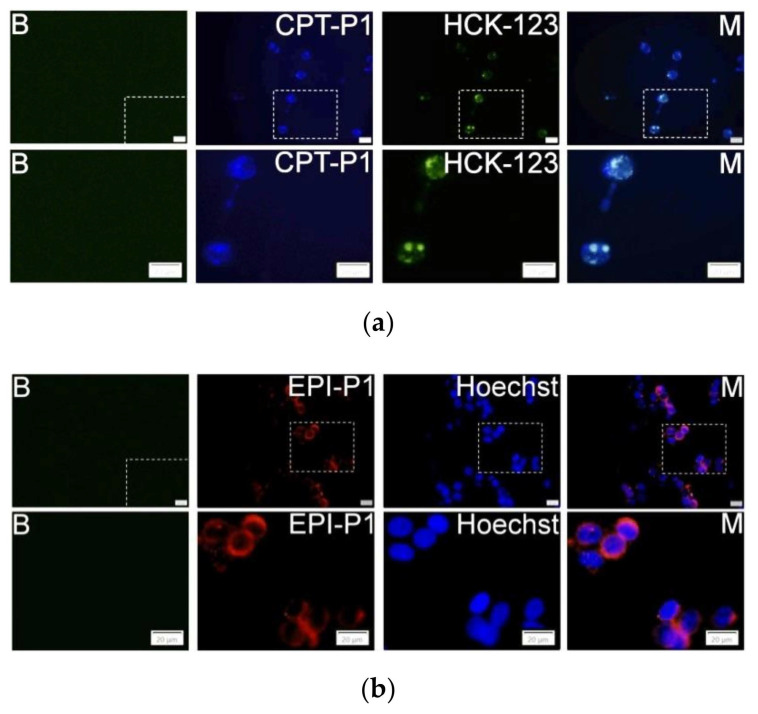
MCF-7 cells fluorescence images and sections of cells without particles and LysoTracker or Hoechst (B). (**a**) Cells with 0.1 mg/mL of CPT-P1 aggregates, 50 nM of LysoTracker Yellow HCK-123 and merged pictures (M); (**b**) Cells with 0.1 mg/mL of EPI-P1 aggregates, 1 µg/mL of Hoechst 33342 and merged pictures (M), scale bars represents 20 µm (see structures in Figure 1).

The uptake and localization of the CPT- and EPI-loaded polyphosphazene nanoaggregates into MCF-7 cancer cells were evaluated by using fluorescence microscopy analysis. Indeed, CPT-P1 (Figure 5a), 1.5CPT-P1, CPT-P2 and CPT-P3 (Supplementary Materials Figures S41–S43) were internalized into the cells (CPT associated blue fluorescence) and accumulated in the lysosomes (LysoTracker HCK-123 associated green fluorescence) [40]. Similarly, EPI-P1 (Figure 5b) and EPI-P2 (Supplementary Materials Figure S44) showed a good uptake into MCF-7 cells (EPI associated red fluorescence). Nuclei were counterstained with Hoechst 33342 (associated blue fluorescence).

**Figure 6:**
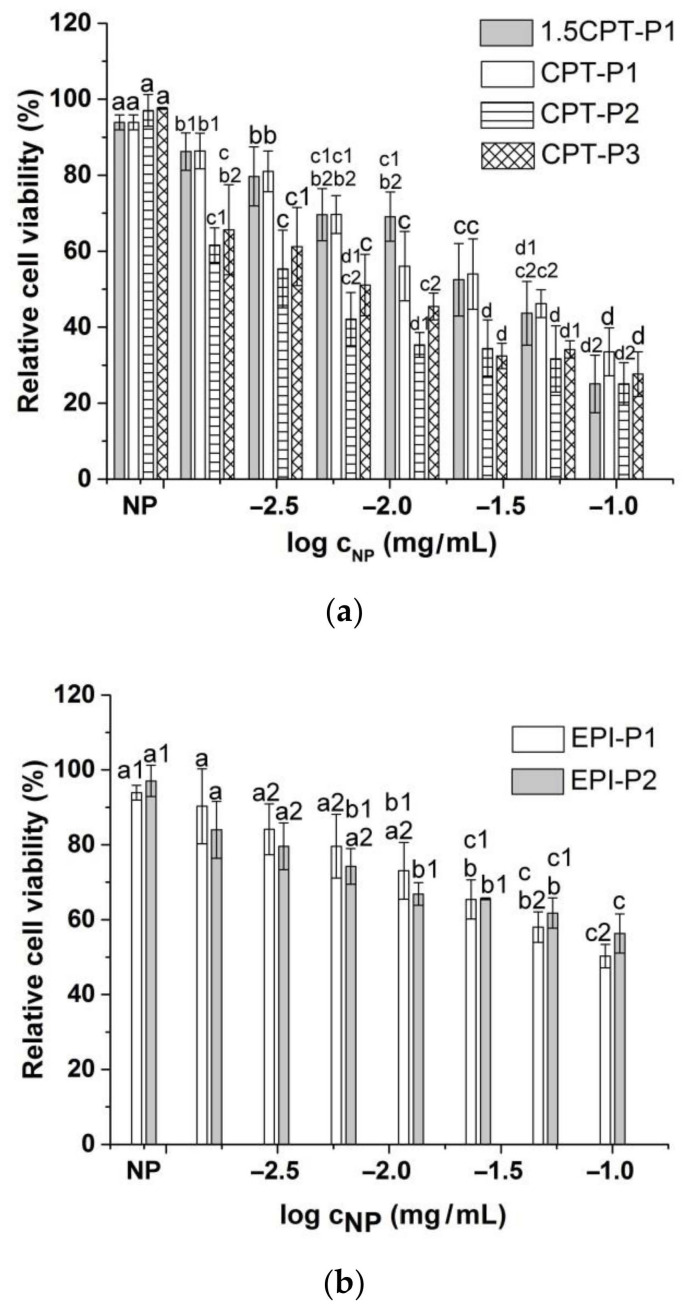
Viability of MCF-7 breast cancer cells treated with (**a**) CPT-loaded polyphosphazene nanoaggregates and (**b**) EPI-loaded polyphosphazene nanoaggregates. NP stands for blank polyphosphazene nanoaggregates at 0.1 mg/mL (see structures in Figure 1). Viability was normalized to the untreated control. Data represent the mean ± standard deviation (*n* = 3). Means with no significant differences are presented with the same letter (*p* > 0.05). Means with significant differences are presented with different letters or the same letter but different numbers following the letter (*p* < 0.05).

Compared to the parent CPT, 1.5CPT-P1 and CPT-P1 with higher CPT contents (13.6% and 10.7%) showed a slightly reduced cell viability of MCF-7 human breast cancer cells at all evaluated concentrations (Figure 6a and Supplementary Materials Figure S45). This might be due to incomplete CPT release from the nanoaggregates observed at day 3 (Figure 4a), associated with hydrophobic interactions and π–π stacking of CPT with the tocopherol core in P1 nanoaggregates. CPT-P2 (9.2 wt% of CPT) with relative cell viability from 25 to 65% and CPT-P3 (9.6 wt% of CPT) with relative cell viability from 28 to 66% exhibited slightly better cytotoxic activity than the parent CPT at all concentrations. On the other hand, EPI-loaded nanoaggregates EPI-P1 (2.4 wt% of EPI) with relative cell viability from 50 to 90% and EPI-P2 (0.3 wt% of EPI) with relative cell viability from 56 to 84% showed a slight to moderate cytotoxic effect when evaluated between 0.1 and 0.0015625 mg/mL of the nanocarriers in solution (Figure 6b). 

Half-maximal inhibitory concentration (IC_50_) of CPT-loaded nanoaggregates against MCF-7 breast cancer cells adjusted to anticancer drug concentration better describes the observed cell viability trend (10^(^^−0.45 ± 0.05)^ µg/mL (1.5CPT-P1) ~10^(^^−0.54 ± 0.06)^ µg/mL (CPT-P1) > 10^(^^−0.95 ± 0.15)^ µg/mL (CPT) > 10^(^^−1.25 ± 0.08)^ µg/mL (CPT-P2) ~10^(^^−1.07 ± 0.08)^ µg/mL (CPT-P3). EPI-P1 nanoaggregates (IC_50_ 10^(^^−0.88 ± 0.07^) µg/mL) appeared to be significantly more effective than the parent EPI (IC_50_ 10^(^^−0.16 ± 0.09^) µg/mL). 

In order to get a better insight into the effect of the different anticancer-drug-loaded nanoaggregates on MCF-7 breast cancer cells after 48 h, Annexin V/propidium iodide (PI) staining was conducted (Supplementary Materials Table S4). CPT-P3 nanoaggregates showed a significant cytotoxic effect at 0.1 mg/mL on MCF-7 cells, with ~66% of dead cells. In contrast, the other CPT-loaded nanoaggregates were slightly more toxic to MCF-7 cells, with ~26% (1.5 CPT-P1, CPT-P1) and 19% (CPT-P2) of dead cells when compared to the parent CPT (9% of dead cells). EPI-loaded nanoaggregates (EPI-P1 and EPI-P2) and the parent EPI exhibited similar moderate toxicity to MCF-7 cancer cells with approximately 13% apoptosis for the parent EPI, compared to ~12% (EPI-P1) and ~20% (EPI-P2) (Supplementary Materials Table S4). Despite their low apoptosis rate, the parent EPI/CPT and the anticancer-drug-loaded nanoaggregates showed a significant reduction in MCF-7 cell viability. Therefore, cell-cycle analyses were performed. Indeed, 1.5CPT-P1 (34% ± 5), EPI-P1 (34% ± 6) and the parent EPI (35% ± 5) showed a slight increase in G2/M phase from 23% ± 6 (control) up to ~34%. Similarly, for CPT-P3, an increase up to 42% ± 7 in G2/M phase was observed. CPT-P1 (69% ± 4), CPT-P2 (67% ± 8) and the parent CPT (64% ± 6) induced a G2/M increase to ~67%. These results indicate a cell-cycle arrest in the G2/M phase (Supplementary Materials Table S5). Interestingly, compared to EPI-P1 and the parent EPI, EPI-P2 induced apoptosis but no MCF-7 cell-cycle arrest.

As the nanoparticles will be administered intravenously to reach their target tissue, their hemocompatibility was determined. It is known that the severe side effects related to the clinical use of CPT derivatives and doxorubicin are associated with their hemolytic activity under physiological conditions [4,5,6]. Therefore, a reduced hemolysis upon application of nanomedicines carrying CPT and EPI is desired.

**Figure 7:**
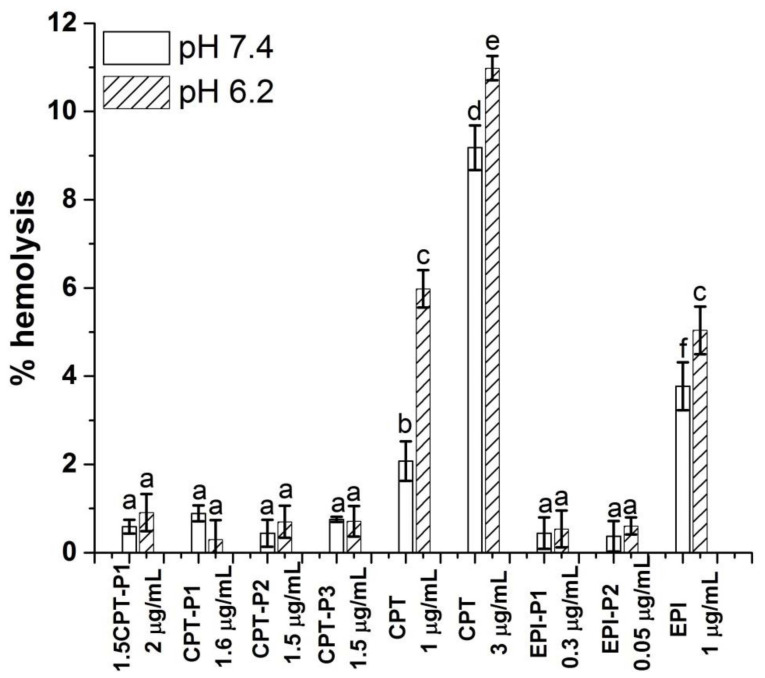
Percentage of hemolysis of RBC treated with CPT- and EPI-loaded polyphosphazene nanoaggregates, parent CPT and EPI in PBS at pH 7.4 and 6.2 (see structures in Figure 1). Data represent the mean ± standard deviation (*n* = 3). The concentrations are expressed in terms of CPT and EPI content. Means with no significant differences are presented with the same letter (*p* > 0.05). Means with significant differences are presented with different letters or the same letter but different numbers following the letter (*p* < 0.05).

Figure 7 shows the hemolytic activity (% hemolysis) of anticancer-drug-loaded nanoaggregates, as well as the parent CPT and EPI, on human red blood cells (RBCs) at pH values of 7.4 and 6.2.

No hemolytic activity was observed for all CPT-loaded nanoaggregates with a CPT content of 1.5 to 2 µg/mL at pH values 7.4 and 6.2 (% hemolysis below 1%) after 24 h. Similarly, EPI-loaded nanoaggregates with an EPI content of 0.34 and 0.045 µg/mL showed hemolysis below 1% at both pH values. Blank nanoparticles at 0.1 mg/mL also resulted innocuous to RBC at pH 7.4 and 6.2 (% hemolysis below 1%) (Supplementary Materials Figure S46). However, the parent CPT exhibited significant hemolytic activity at pH 7.4 (2%) and pH 6.2 (6%) at 1 µg/mL of CPT with an increasing hemolysis from 9 to 11% respectively at 3 µg/mL of CPT. The hemolytic activity of the parent EPI at 1 µg/mL was even higher at pH 7.4 (4%) and 5% at pH 6.2. 

### 3.5. Spheroid Uptake and Cytotoxic Activity

**Figure 8:**
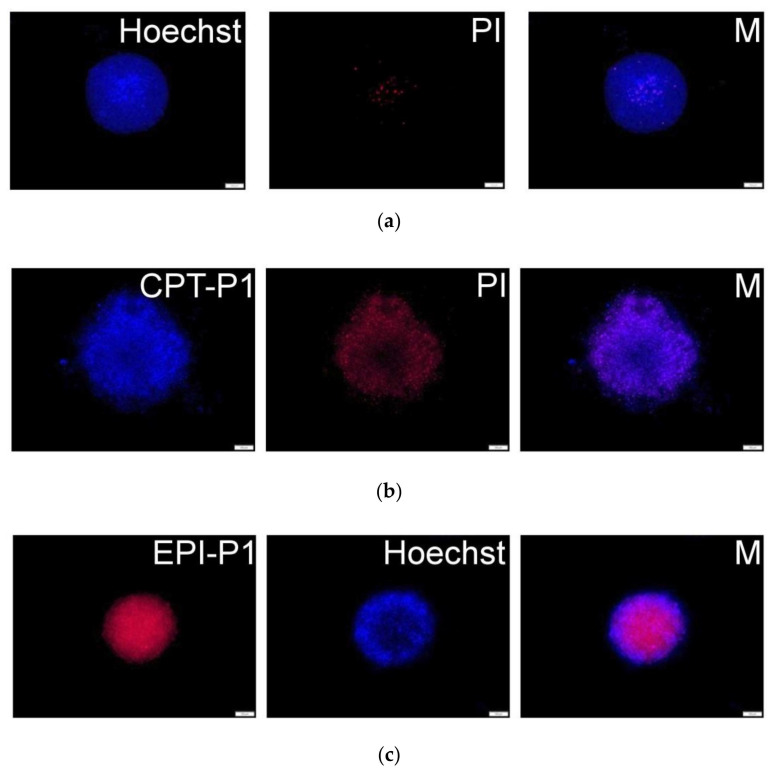
(**a**) MCF-7 spheroid fluorescence images without particles and propidium iodide or Hoechst staining. (**b**) Spheroids with 0.1 mg/mL of CPT-P1 aggregates, propidium iodide (PI) and merged pictures (M). (**c**) Spheroids with 0.1 mg/mL of EPI-P1 aggregates, Hoechst 33,342 and merged pictures (M). Scale bars represent 100 µm (see structures in Figure 1).

To obtain a 3D-like architecture, MCF-7 cells were grown as spheroids to evaluate the uptake and cytotoxicity of synthesized CPT- and EPI-loaded nanoaggregates. Fluorescence microscopy images (Figure 8b and Supplementary Materials Figure S47) showed a good uptake of CPT-loaded nanoaggregates into MCF-7 spheroids (CPT associated blue fluorescence). Further, extensive cell death (propidium iodide related red fluorescence) was observed after 72 h. Similarly, for EPI-P1 (Figure 8c) and EPI-P2 (Supplementary Materials Figure S48) substantial uptake into MCF-7 spheroids (EPI associated red fluorescence) could be detected. Nuclei were counterstained with Hoechst 33,342 (associated blue fluorescence).

Additionally, after treatment with EPI-loaded nanoaggregates, extensive cell death (irregular spheroids borderline compared to non-treated control) could be detected in MCF-7 spheroids after 72 h (Supplementary Materials Figures S49–S51).

Cell viability of MCF-7 spheroids was evaluated after 72 h of treatment with anticancer-drug-loaded polyphosphazene aggregates or the parent CPT/EPI. Half-maximal inhibitory concentration of CPT-loaded aggregates to MCF-7 spheroids, adjusted to CPT or EPI concentration, showed significantly reduced cell viability for all CPT-loaded aggregates compared to the parent CPT. (10^(0.2 ± 0.2)^ µg/mL (CPT) > 10^(^^−0.17 ± 0.06)^ µg/mL (CPT-P3) > 10^(^^−0.7 ± 0.1)^ µg/mL (1.5CPT-P1) ~10^(^^−0.46 ± 0.05)^ µg/mL (CPT-P1) ~10^(^^−0.53 ± 0.07)^ µg/mL (CPT-P2). EPI-P1 nanoaggregates (IC_50_ 10^(^^−0.52 ± 0.03)^ µg/mL) also appeared significantly more effective than the parent EPI (IC_50_ 10^(0.75 ± 0.07)^ µg/mL). 

It is assumed that CPT- and EPI-loaded polyphosphazene nanoaggregates might accumulate in cancer tissues due to combination of passive diffusion and EPR effect [36,37,41]. Moreover, 158 nm CPT-P2 and 194 nm CPT-P3 nanoaggregates might also exhibit extended circulation times and moderated reticuloendothelial system (RES) clearance [37]. Once CPT-P2 or CPT-P3 nanoaggregates are inside the cancer cells, a co-delivery of CPT cargo and tocopherol or testosterone might occur due to enzyme-promoted and acidic hydrolysis of ester bond in the tocopherol- or testosterone-glycine moiety. This co-delivery of tocopherol or testosterone with the anticancer drugs might enhance the therapeutic effect and reduce side effects, due to the reported antioxidant and potential antitumor activity of the vitamin E and testosterone.

On the other hand, blank P1–P3 nanoaggregates showed almost no toxicity to primary human lung fibroblasts within the entire concentration range evaluated (from 0.0015625 to 0.1 mg/mL), with observed relative cell viabilities from 80 to 100% for P1, ~90 to 100% for P2 and approximately 100% for P3 (Supplementary Materials Figure S52).

## 4. Conclusions

Tocopherol and testosterone functionalized polyphosphazenes were synthesized via living cationic polymerization of the monomer trichlorophosphoranimine (Cl_3_P = N–Si(CH_3_)_3_) and subsequent post-polymerization functionalization. These polyphosphazenes self-aggregated in aqueous medium and were loaded with the anticancer drugs CPT and EPI, respectively. CPT and EPI were slowly released in simulated physiological conditions (PBS, pH 7.4, 37 °C) during 8 days, with almost constant release rate during the first 8 h. Fluorescence microscopy imaging confirmed good cellular uptake of CPT- and EPI-loaded nanoaggregates into the MCF-7 cell line and spheroids, as well as lysosomal accumulation of CPT-loaded nanoaggregates. CPT- and EPI-loaded nanocarriers were similarly or more toxic to MCF-7 human breast cancer cells, as compared to the parent anticancer drugs. Furthermore, CPT- and EPI-loaded aggregates (except EPI-P2) caused significant cell-cycle arrest in the G2/M phase and induced significant apoptosis of MCF-7 cells. These CPT- and EPI-loaded nanocarriers exhibited lower IC_50_ values than the parent anticancer drugs in MCF-7 spheroids. Additionally, the absence of hemolytic activity of the anticancer-drug-loaded polyphosphazene aggregates makes them good candidates for chemotherapy.

## Figures and Tables

**Table 1:** Composition, yield and molecular weight of polymers P1–P3.

Sample	DP ^1^	ToT:M1000 ^2^	Y ^3^ (%)	M_n_^Theor^(kg/mol) ^4^	M_n_^exp^(kg/mol) ^5^	M_w_/M_n_^5^
P1	24	1:1	56	39	14	1.35
P2	20	1:3	44	45	19	1.24
P3	21	1:3	48	43	17	1.48

^1^ Degree of polymerization estimated by ^1^H NMR. ^2^ Tocopherol or testosterone to Jeffamine M1000 ratio feeding composition. ^3^ Yield of the reaction. ^4^ Theoretical number average molecular weight. ^5^ Number average molecular weight and polydispersity measured by GPC against polystyrene standards.

**Table 2:** Hydrodynamic sizes, average diameters by AFM of dried nanoaggregates P1–P3, anticancer drug weight contents (wt%) and encapsulation efficiency (EE%) of the anticancer drugs.

Sample	*d*_h_^1^ nm (PDI)	*d*_h_^2^ nm (PDI)	*d*_AFM_^3^ nm	wt% ^4^	EE% ^5^
P1	21 ± 1 ^a^ (0.29)	22 ± 3 ^a^ (0.30)	27 ± 9	-	-
P2	108 ± 8 (0.58)	127 ± 3 (0.42)	42 ± 5	-	-
P3	134 ± 2 (0.55)	96 ± 2 (0.61)	58 ± 6	-	-
1.5CPT-P1	-	198 ± 11 ^b^ (0.54)	-	13.6	83
CPT-P1	-	142 ± 1 (0.69)	-	10.7	94
CPT-P2	-	158 ± 3 (0.54)	-	9.2	91
CPT-P3	-	194 ± 5 ^b^ (0.29)	-	9.6	87
EPI-P1	-	250 ± 9 ^c^ (0.65)	-	2.4	23
EPI-P2	-	253 ± 4 ^c^ (0.52)	-	0.3	3

^1^ Average hydrodynamic diameter (*d*_h_) in water by DLS. ^2^ Average hydrodynamic diameter (*d*_h_) in PBS by DLS. ^3^ Diameter of dried nanoaggregates by AFM (*d*_AFM_). ^4^ Anticancer drug weight contents (wt%). ^5^ Encapsulation efficiency ($\mathrm{EE}\%=\left(\frac{\mathrm{weight}\;\mathrm{of}\;\mathrm{CPT}\;\mathrm{or}\;\mathrm{EPI}\;\mathrm{loaded}\;\mathrm{in}\;\mathrm{particles}}{\mathrm{weight}\;\mathrm{of}\;\mathrm{feeding}\;\mathrm{CPT}\;\mathrm{or}\;\mathrm{EPI}}\right)\times 100$. Data represent the mean ± standard deviation (*n* = 3). Means with no signif- icant differences are presented with the same letter (*p* > 0.05). Means with significant differences are presented with different letters or the same letter but different numbers following the letter (*p* < 0.05).

## Data Availability

Data available in Supplementary Materials.

## Supplementary Materials

The following are available online at https://www.mdpi.com/article/10.3390/pharmaceutics14010169/s1. Table S1: Linear fitting parameters of in vitro CPT and EPI release profiles of CPT- and EPI-loaded polyphosphazene nanoaggregates up to 8 h (intercept 0, slope k and adjusted R-Square) in PBS (pH 7.4) at 37 °C. Table S2: SWeibull2 fitting parameters of cumulative release (%) vs. time (hours) of CPT- and EPI-loaded polyphosphazene nanoaggregates up to 202 h (cumulative release (%) = a − (a − b)*exp(−(k*time(hours))^d^)) in PBS (pH 7.4) at 37 °C. Table S3: Linear fitting parameters of log(cumulative release (%)) vs. log(time (hours)) of CPT- and EPI-loaded polyphosphazene nanoaggregates up to 100 h (intercept m, slope k and adjusted R-Square) in PBS (pH 7.4) at 37 °C. Table S4: MCF-7 cell cytotoxicity at control and 0.1 mg/mL of CPT- and EPI-loaded nanoaggregates and parent anticancer drugs determined by Annexin V and propidium iodide. Table S5: MCF-7 cell-cycle-arrest parameters at control and 0.1 mg/mL of CPT- and EPI-loaded nanoaggregates and parent anticancer drugs. Synthesis of materials, monomer and polymers P1–P3. Characterization data for materials and polymers P1–P3. Figure S1: HPLC chromatograms at 370 nm of 1.5CPT-P1 at 0.0367 mg/mL, CPT-P1 at 0.064 mg/mL, CPT-P2 at 0.017 mg/mL, CPT-P3 at 0.017 mg/mL in PBS (see structures in Figure 1 in manuscript). Figure S2: Calibration curve of CPT in PBS (pH 7.4) obtained from HPLC chromatograms at 254 nm. Figure S3: Calibration curve of CPT in PBS (pH 7.4) obtained from HPLC chromatograms at 370 nm. Figure S4: HPLC chromatograms at 254 nm of EPI-P1 at 0.053 mg/mL and EPI/P2 at 0.017 mg/mL in PBS (see structures in Figure 1 in manuscript). Figure S5: Calibration curve of EPI in PBS (pH 7.4) obtained from HPLC chromatograms at 254 nm. Figure S6. Calibration curve of EPI in PBS (pH 7.4) obtained from HPLC chromatograms at 475 nm. Figure S7: Calibration curve of testosterone in PBS (pH 7.4) (testosterone $\varepsilon_{249}^{PBS\;}$ = 17,594 M^−1^ cm^−1^) (see structures in Figure 1 in manuscript). Figure S8: Calibration curve of testosterone in PBS (pH 6.0) (testosterone $\varepsilon_{249}^{PBS}\;$ = 16,613 M^−1^ cm^−1^) (see structures in Figure 1 in manuscript). Figure S9: Calibration curve of DL-α-tocopherol in PBS (pH 7.4) (tocopherol $\varepsilon_{290}^{PBS}$ = 8960 M^−1^ cm^−1^) (see structures in Figure 1 in manuscript). Figure S10: Calibration curve of DL-α-tocopherol in PBS (pH 6.0) (tocopherol $\varepsilon_{290}^{PBS}$ = 10,768 M^−1^ cm^−1^) (see structures in Figure 1 in manuscript). Figure S11: FTIR spectra of tocopherol-glycine-NH_2_ (1) (see structures in Figure 1 in manuscript). Figure S12: FTIR spectra of testosterone-glycine-NH_2_ (2) (see structures in Figure 1 in manuscript). Figure S13: ^1^H NMR (300 MHz, 298 K, CDCl_3_) spectrum of tocopherol-glycine-NH_2_ (1) (see structures in Figure 1 in manuscript), Figure S14: ^1^H NMR (300 MHz, 298 K, CDCl_3_) spectrum of testosterone-glycine-NH_2_ (2) (see structures in Figure 1 in manuscript). Figure S15: APT ^13^C NMR (75 MHz, 298 K, CDCl_3_) spectrum of tocopherol-glycine-NH_2_ (1) (see structures in Figure 1 in manuscript). Figure S16: APT ^13^C NMR (75 MHz, 298 K, CDCl_3_) spectrum of testosterone-glycine-NH_2_ (2) (see structures in Figure 1 in manuscript). Figure S17: ^31^P{^1^H} NMR (121 MHz, 298 K, CDCl_3_) spectrum of monomer trichlorophosphoranimine (Cl_3_P = N − Si(CH_3_)_3_). Figure S18: ^31^P{^1^H} NMR (121 MHz, 298 K, CDCl_3_) spectrum of polydichlorophosphazene. Figure S19: ^1^H NMR (300 MHz, 298 K, CDCl_3_) spectrum of monomer trichlorophosphoranimine (Cl_3_P = N − Si(CH_3_)_3_). Figure S20: ^1^H NMR (300 MHz, 298 K, CDCl_3_) spectrum of polydichlorophosphazene. Figure S21: ^1^H NMR (300 MHz, 298 K, CDCl_3_) spectrum of polymer P2. DP was calculated as $\mathrm{DP}=\frac{15}{I_{{\mathrm{Ph}}_{3}P-}}=\frac{15}{\mathrm{Ia}}$ (see structures in Figure 1 in manuscript). Figure S22: ^1^H NMR (300 MHz, 298 K and CDCl_3_) spectrum of polymer P3. DP was calculated as $\mathrm{DP}=\frac{15}{I_{{\mathrm{Ph}}_{3}P-}}=\frac{15}{\mathrm{Ia}}$ (see structures in Figure 1 in manuscript). Figure S23: APT ^13^C NMR (75 MHz, 298 K, CDCl_3_) spectrum of polymer P1 (see structures in Figure 1 in manuscript). Figure S24: GPC chromatogram of polymer P1 (see structures in Figure 1 in manuscript). Figure S25: GPC chromatogram of polymer P2 (see structures in Figure 1 in manuscript). Figure S26: GPC chromatogram of polymer P3 (see structures in Figure 1 in manuscript). Figure S27: Calorimetry differential scanning curve of polymer P1 (see structures in Figure 1 in manuscript). Figure S28: Calorimetry differential scanning curve of polymer P2 (see structures in Figure 1 in manuscript). Figure S29: Calorimetry differential scanning curve of polymer P3 (see structures in Figure 1 in manuscript). Figure S30: TGA curve of P1 (see structures in Figure 1 in manuscript). Figure S31: TGA curve of P2 (see structures in Figure 1 in manuscript). Figure S32: TGA curve of P3 (see structures in Figure 1 in manuscript). Figure S33: SEM micrographs of P1 and P2 dried nanoaggregates at 20,000× magnification (see structures in Figure 1 in manuscript). Figure S34: Linear fitting of in vitro CPT release profiles of CPT-loaded polyphosphazene nanoaggregates up to 8 h in PBS (pH 7.4) at 37 °C (see structures in Figure 1 in manuscript). Figure S35: Linear fitting of in vitro EPI release profiles of EPI-loaded polyphosphazene nanoaggregates up to 8 h in PBS (pH 7.4) at 37 °C (see structures in Figure 1 in manuscript). Figure S36: Linear fitting of log(cumulative release (%)) vs. log(time (hours)) of CPT-loaded polyphosphazene nanoaggregates up to 100 h in PBS (pH 7.4) at 37 °C (see structures in Figure 1 in manuscript). Figure S37: Linear fitting of log(cumulative release (%)) vs. log(time (hours)) of EPI-loaded polyphosphazene nanoaggregates up to 24 h in PBS (pH 7.4) at 37 °C (see structures in Figure 1in manuscript). Figure S38: In vitro release profiles of tocopherol from P1 aggregates in PBS (pH 7.4 and 6.0) at 37 °C, adjusted to a SWeibull2 function (see structures in Figure 1 in manuscript). Figure S39: In vitro release profiles of tocopherol from P2 aggregates in PBS (pH 7.4 and 6.0) at 37 °C, adjusted to a SWeibull2 function (see structures in Figure 1 in manuscript). Figure S40: In vitro release profiles of testosterone from P3 aggregates in PBS (pH 7.4 and 6.0) at 37 °C, adjusted to a SWeibull2 function (see structures in Figure 1 in manuscript). Figure S41: MCF-7 cells’ fluorescence images and slices of cells without particles and LysoTracker (B). Cells with 0.1 mg/mL of 1.5CPT-P1 aggregates, 50 nM of LysoTracker Yellow HCK-123 and merged pictures (M). Scale bars represent 20 µm (see structures in Figure 1 in manuscript). Figure S42: MCF-7 cells’ fluorescence images and slices of cells without particles and LysoTracker (B). (a) Cells with 0.1 mg/mL of CPT-P2 aggregates, 50 nM of LysoTracker Yellow HCK-123 and merged pictures (M). Scale bars represent 20 µm (see structures in Figure 1 in manuscript). Figure S43: MCF-7 cells’ fluorescence images and slices of cells without particles and LysoTracker (B). (a) Cells with 0.1 mg/mL of CPT-P3 aggregates, 50 nM of LysoTracker Yellow HCK-123 and merged pictures (M). Scale bars represent 20 µm (see structures in Figure 1 in manuscript). Figure S44: MCF-7 cells’ fluorescence images and slices of cells without particles and Hoechst (B). Cells with 0.1 mg/mL of EPI-P2 aggregates, 1 µg/mL of Hoechst 33342 and merged pictures (M). Scale bars represent 20 µm (see structures in Figure 1 in manuscript). Figure S45: Relative cell viability of MCF-7 breast cancer cells treated with parent CPT or EPI. Data represent the mean ± standard deviation (*n* = 3). Means with no significant differences are presented with the same letter (*p* > 0.05). Means with significant differences are presented with different letters or the same letter but different numbers following the letter (*p* < 0.05). Figure S46: Percentage of hemolysis of RBC treated with 0.1 mg/mL of blank P1–P3 polyphosphazene nanoaggregates in PBS at pH 7.4 and 6.2 (see structures in Figure 1 in manuscript). Data represent the mean ± standard deviation (*n* = 3). Means with no significant differences are presented with the same letter (*p* > 0.05). Means with significant differences are presented with different letters or the same letter but different numbers following the letter (*p* < 0.05). Figure S47: MCF-7 spheroid fluorescence images with 0.1 mg/mL of CPT or CPT-loaded aggregates, propidium iodide (PI) and merged pictures (M). Scale bars represents 100 µm (see structures in Figure 1 in manuscript). Figure S48: MCF-7 spheroid fluorescence images with 0.1 mg/mL of EPI or EPI-P2 aggregates, Hoechst 33342 and merged pictures (M). Scale bars represent 100 µm (see structures in Figure 1 in manuscript). Figure S49: MCF-7 spheroids’ bright field images without particles (B) or with 0.1 mg/mL of CPT or CPT-P1 nanoaggregates at 0 h (a) and 72 h (b). Scale bars represent 100 µm (see structures in Figure 1 in manuscript). Figure S50: MCF-7 spheroids’ bright field images with 0.1 mg/mL of CPT-loaded nanoaggregates at 0 h (a) and 72 h (b). Scale bars represent 100 µm (see structures in Figure 1 in manuscript). Figure S51: MCF-7 spheroids’ bright field images with 0.1 mg/mL of EPI or EPI-loaded nanoaggregates. Scale bars represent 100 µm (see structures in Figure 1 in manuscript). Figure S52: Relative cell viability of primary human lung fibroblasts treated with blank P1–P3 polyphosphazene nanoaggregates (see structures in Figure 1 in manuscript). Data represent the mean ± standard deviation (*n* = 3). Means with no significant differences are presented with the same letter (*p* > 0.05). Means with significant differences are presented with different letters or the same letter but different numbers following the letter (*p* < 0.05).
