# Supplementary material for: Polyphosphazene-Based Nanocarriers for the Release of Camptothecin and Epirubicin
Source: Pharmaceutics. 2022 Jan 11;14(1):169. doi: 10.3390/pharmaceutics14010169 (PMC8781282; doi:10.3390/pharmaceutics14010169)
Supplement: Supplementary file 1 [file pharmaceutics-14-00169-s001.zip › pharmaceutics-1422950-supplementary.pdf]

pharmaceutics

Supplementary Materials: Polyphosphazene-Based Nanocarriers for the Release of Camptothecin and Epirubicin

Javier Pérez Quiñones, Cornelia Roschger, Aitziber Iturmendi, Helena Henke, Andreas Zierer, Carlos Peniche-Covas and Oliver Brüggemann

Table S1. Linear fitting parameters of in vitro CPT and EPI release profiles of CPT- and EPI-loaded polyphosphazene nanoaggregates up to 8 h (intercept 0, slope k and adjusted R-Square) in PBS (pH 7.4) at 37 °C.

Samples	k	Adjusted R-Square
1.5CPT-P1	0.60 ± 0.01	0.9970
CPT-P1	0.54 ± 0.02	0.9938
CPT-P2	0.79 ± 0.02	0.9967
CPT-P3	1.09 ± 0.02	0.9982
EPI-P1	1.86 ± 0.05	0.9966
EPI-P2	1.22 ± 0.09	0.9734

Table S2. SWeibull2 fitting parameters of cumulative release (%) vs. time (hours) of CPT- and EPI-loaded polyphosphazene nanoaggregates up to 202 h (cumulative release (%) = a − (a − b)*exp(−(k*time(hours))^{d})) in PBS (pH 7.4) at 37 °C.

Samples	A	b	k	d	Adjusted R-Square
1.5CPT-P1	120 ± 20[a]	0.0 ± 0.2[b1]	0.006 ± 0.001[c1]	1.02 ± 0.05[d]	0.9989
CPT-P1	55 ± 2	0.0 ± 0.3[b1]	0.025 ± 0.003	1.10 ± 0.07[d]	0.9966
CPT-P2	105 ± 7[a]	0.0 ± 0.2[b1]	0.010 ± 0.001[c2]	1.08 ± 0.04[d]	0.9975
CPT-P3	110 ± 30[a]	0.0 ± 0.3[b1]	0.006 ± 0.002[c1]	1.05 ± 0.08[d]	0.9915
EPI-P1	41.7 ± 0.4	2.5 ± 0.3[b2]	0.056 ± 0.003	1.30 ± 0.09	0.9986
EPI-P2	120 ± 40[a]	0 ± 4[b]	0.008 ± 0.005[c]	0.8 ± 0.2[d]	0.9898

Means with no significant differences are presented with the same letter ($p > 0.05$). Means with significant differences are presented with different letters or the same letter but different numbers following the letter ($p < 0.05$).

Table S3. Linear fitting parameters of log(cumulative release (%)) vs. log(time (hours)) of CPT- and EPI-loaded polyphosphazene nanoaggregates up to 100 h (intercept m, slope k and adjusted R-Square) in PBS (pH 7.4) at 37 °C.

Samples	m	k	Adjusted R-Square
1.5CPT-P1	−0.25 ± 0.03[a]	1.00 ± 0.02[c1]	0.9969
CPT-P1	−0.32 ± 0.04[a]	1.03 ± 0.03[c1]	0.9951
CPT-P2	−0.12 ± 0.04	0.99 ± 0.03[c1]	0.9948
CPT-P3	0.12 ± 0.06[b]	0.85 ± 0.04[c2,d]	0.9812
EPI-P1	0.43 ± 0.02	0.79 ± 0.02[d]	0.9963
EPI-P2	0.18 ± 0.07[b]	0.95 ± 0.07[c]	0.9829

Means with no significant differences are presented with the same letter ($p > 0.05$). Means with significant differences are presented with different letters or the same letter but different numbers following the letter ($p < 0.05$).

Table S4. MCF-7 cell cytotoxicity at control and 0.1 mg/mL of CPT- and EPI-loaded nanoaggregates, and parent anticancer drugs determined by Annexin V and propidium iodide.

Sample	Alive (%)	Necrosis (%)	Early apoptosis (%)	Late apoptosis (%)
Control	95 ± 3^{a}	1.9 ± 0.6^{d1}	2 ± 2^{d1}	1.1 ± 0.6^{d1}
1.5CPT-P1	75.7 ± 0.6^{b1}	$7 \pm 3^{d2,e}$	$11 \pm 6^{d2,e,f}$	$7 \pm 2^{d2,e}$
CPT-P1	73 ± 2^{b2}	6 ± 8^{d}	$16 \pm 15^{d,f}$	4 ± 5^{d}
CPT-P2	81 ± 1^{b3}	$3 \pm 3^{d1,e}$	$14 \pm 6^{d2,f}$	2 ± 2^{d1}
CPT-P3	34 ± 4^{c}	6 ± 11^{d}	$38 \pm 29^{c,d2,f}$	$41 \pm 25^{c,f}$
CPT	91 ± 2^{a}	1.5 ± 0.7^{d1}	$6 \pm 1^{d4,e}$	1.5 ± 0.3^{d1}
EPI-P1	85 ± 3^{b4}	3.2 ± 0.9^{d1}	$10 \pm 4^{d2,e}$	2.0 ± 0.3^{d1}
EPI-P2	78 ± 2^{b1}	2.3 ± 0.2^{d1}	18 ± 2^{f}	1.6 ± 0.2^{d1}
EPI	75 ± 10^{b}	$3 \pm 5^{d1,e}$	$8 \pm 9^{d,f}$	$6 \pm 2^{d2,e}$

Means with no significant differences are presented with the same letter ($p > 0.05$). Means with significant differences are presented with different letters or the same letter but different numbers following the letter ($p < 0.05$).

Table S5. MCF-7 cell-cycle-arrest parameters at control and 0.1 mg/mL of CPT- and EPI-loaded nanoaggregates and parent anticancer drugs.

Sample	G0, G1 (%)	S (%)	G2, M (%)
Control	69 ± 7^{a}	7 ± 1^{d1}	23 ± 6^{e}
1.5CPT-P1	61 ± 5^{a}	6 ± 8^{d}	34 ± 5^{c}
CPT-P1	27 ± 7^{b}	4 ± 1^{d2}	69 ± 4^{g}
CPT-P2	30 ± 7^{b}	2.1 ± 0.4^{d3}	67 ± 8^{g}
CPT-P3	43 ± 4^{c}	15 ± 8^{d1}	42 ± 7^{c}
CPT	31 ± 7^{b}	4.7 ± 0.9^{d2}	64 ± 6^{g}
EPI-P1	63 ± 4^{a}	4 ± 5^{d}	34 ± 6^{c}
EPI-P2	67 ± 4^{a}	$7 \pm 3^{d1,d2}$	26 ± 4^{e}
EPI	63 ± 5^{a}	2.5 ± 0.2^{d3}	35 ± 5^{c}

Means with no significant differences are presented with the same letter ($p > 0.05$). Means with significant differences are presented with different letters or the same letter but different numbers following the letter ($p < 0.05$).

Synthesis of materials, monomer and polymers P1–P3

Synthesis of tocopherol-glycine-NH_2 (1) and testosterone-glycine-NH_2 (2): Boc-protected amino acid Boc-Gly-OH (85 mg, 0.49 mmol), 4-(dimethylamino)pyridine (59 mg, 0.48 mmol) and *N,N′*-dicyclohexylcarbodiimide (124 mg, 0.60 mmol) were dissolved in 15 mL CH_2Cl_2 and stirred at room temperature for 2 h. The reaction mixture was added to a solution of tocopherol (207 mg, 0.48 mmol) or testosterone (139 mg, 0.48 mmol) in 10 mL CH_2Cl_2 and stirred for 48 h. The precipitated *N,N′*-dicyclohexylurea was removed by filtration, and the filtrated material was extracted with 10% NH_4Cl aqueous solution (2 × 15 mL), with 5% $NaHCO_3$ aqueous solution (2 × 15 mL) and saturated NaCl solution (1 × 15 mL). The organic phase was dried over $MgSO_4$, filtered and removed under reduced pressure to yield tocopherol-glycine-Boc as a white solid (212 mg, yield 75%) or testosterone-glycine-Boc as a slightly yellow solid (165 mg, yield 77%). Then, tocopherol-glycine-Boc (212 mg, 0.36 mmol) or testosterone-glycine-Boc (165 mg, 0.37 mmol) was dissolved in 10 mL of CH_2Cl_2. CF_3COOH (1 mL, 13.10 mmol) was added dropwise to the tocopherol-glycine-Boc solution and stirred at room temperature overnight. The excess of CF_3COOH and the solvent were removed under reduced pressure; CH_2Cl_2 was added and removed under reduced pressure twice. The remnant solid was dissolved again in 30 mL of CH_2Cl_2 and washed with 5% $NaHCO_3$ aqueous solution (2 × 15 mL) and saturated NaCl solution (1 × 15 mL) and dried over $MgSO_4$. The CH_2Cl_2 was removed under reduced pressure to obtain tocopherol-glycine-NH_2 (1) as a white powder (131 mg, yield 75%), or testosterone-glycine-NH_2 (2) as a yellow powder (74.4 mg, yield 58%).

Synthesis of monomer trichlorophosphoranimine ($Cl_3P=N–Si(CH_3)_3$): Lithium bis(trimethylsilyl)amide ($LiN(Si(CH_3)_3)_2$) (25.00 g, 149.41 mmol) was dissolved in 500 mL of anhydrous Et_2O under Ar atmosphere, cooled to 0–4 °C with ice bath and stirred for 0.5 h. A total of 13.07 mL of PCl_3 (20.52 g, 149.41 mmol) was slowly added dropwise with a 20 mL syringe, while the reaction mixture was stirred at 0–4 °C. Then, the reaction mixture was stirred at room temperature for 1 h. The solution was cooled to 0–4 °C with an ice bath for a second time, 12.1 mL of SO_2Cl_2 (20.17 g, 149.41 mmol) was added dropwise and the mixture was stirred for 1 h at 0–4 °C. Then, the reaction mixture was fast filtered over Celite, and Et_2O was removed under reduced pressure at room temperature. The purification of the product was carried out via vacuum distillation with a Büchi glass oven (Büchi Labortechnik, Switzerland) at 40 °C, under reduced pressure of 4 mbar, to obtain $Cl_3P=N–Si(CH_3)_3$ as a colorless liquid. The monomer was stored under Ar atmosphere at -35 °C in the glovebox (18.00 g, yield 54%). ^1H NMR (300 MHz, $CDCl_3$): δ = 0.16 (s, 9H) ppm; $^{31}P\{^1H\}$ NMR (121 MHz, $CDCl_3$): δ = -54.2 ppm.

Synthesis of the polymer P1: $(C_6H_5)_3PCl_2$ (3.27 mg, 0.01 mmol) and $Cl_3P=N–Si(CH_3)_3$ (55.00 mg, 0.25 mmol) were dissolved in 1 mL of anhydrous CH_2Cl_2 and stirred at room temperature overnight. Then, the obtained poly(dichloro)phosphazene (yield quantitative) was transferred to another flask with tocopherol-glycine-NH_2 (1) (120.0 mg, 0.25 mmol) and excess of Et_3N (72.6 mg, 0.72 mmol) in 10 mL of anhydrous THF and stirred at room temperature for 24 h. Afterwards, the second post-polymerization functionalization was carried out with Jeffamine M1000. An excess of Jeffamine M1000 (344.0 mg, 0.34 mmol) and Et_3N (108.9 mg, 1.08 mmol) were added to the mixture and allowed to react another 24 h. Afterwards, the solvent was removed under reduced pressure, dispersed in 15 mL of absolute EtOH and the product was purified by dialysis against deionized water (3 L, 2 times, 8 h) and EtOH (750 mL, 8 times, 4 days). The EtOH was removed with N_2 flow, and the polymer was further dried under vacuum to give the polymer P1 as a slightly brown waxy solid (212.0 mg, yield 56%). Polymer P2 was obtained also as a slightly brown waxy solid (200 mg, yield 45%), while polymer P3 appeared as a colorless waxy solid (130 mg, yield 48%).

Characterization data for materials and polymers P1–P3

Tocopherol-glycine-NH_2 (1): yield 131 mg (75%). ATR–FTIR (solid) υ max: 3323 (N–H), 2927 (C–H), 1760 (C=O), 1624 (N-H bend), 1571 (N-H bend), 1449 (C–H bend), 1243 (C–O–C) cm^{-1}; ^1H NMR (300 MHz, 298 K, $CDCl_3$): δ = 0.85 (t, 12H, 4′C*H*$_3$- + 8′C*H*$_3$- + 12′C*H*$_3$-), 1.96-2.11 (m, 9H, 5C*H*$_3$- + 7C*H*$_3$- + 8C*H*$_3$-), 2.59 (t, 2H, H4), 3.48 (m, 1H, NH2–C***H***2–COO–), 3.76 (s, 1H, NH2–C***H***2–COO–) ppm; ^{13}C NMR (75 MHz, $CDCl_3$): δ = 11.4 (5CH_3-), 12.0 (8CH_3-), 12.3 (7CH_3-), 19.8 (4′CH_3-), 19.9 (8′CH_3-), 20.7 (C4), 21.2 (C2′), 22.8 (12′C*H*$_3$-), 22.9 (12′C*H*$_3$-), 23.9 (2CH_3-), 24.6 (C6′), 25.0 (C10′), 28.1 (C12′), 32.8 (C4′), 32.9 (C8′), 34.1 (C3), 37.4 (C7′), 37.5 (C5′), 37.6 (C9′), 37.7 (C3′), 39.5 (C1′ + C11′), 43.9 (NH2–*C*H2–COO–), 74.7 (C2), 117.6 (C9), 118.7 (C5), 121.3 (C7), 122.7 (C8), 144.7 (C9), 145.7 (C10), 149.7 (C6), 173.1 (*C*=O, glycine) ppm [1]. ESI–MS $[M+H]^+$ m/z: 488.4100; calculated $[M+H]^+$ m/z: 488.4098.

Testosterone-glycine-NH_2 (2): yield 74.4 mg (58%). ATR–FTIR (solid) υ max: 3324 (N–H), 2931 (C–H), 1733 (C=O), 1671 (C=O, ketone C3 of testosterone), 1625 (N-H bend), 1574 (N-H bend), 1448 (C–H bend), 1183 (C–O–C) cm^{-1}; ^1H NMR (300 MHz, 298 K, $CDCl_3$): δ = 0.82 (s, 3H, H18), 1.18 (s, 3H, H19), 3.41-3.87 (m, 2H, NH2–C***H***2–COO–), 5.72 (s, 1H, H4) ppm [2]; ^{13}C NMR (75 MHz, $CDCl_3$): δ = 12.2 (C18), 17.6 (C19), 20.7 (C11), 23.7 (C15), 27.7 (C16), 31.6 (C7), 32.9 (C6), 34.1 (C2), 35.5 (C8), 35.9 (C1), 36.8 (C12), 38.8 (C10), 42.7 (C13), 44.2 (NH2–*C*H2–COO–), 50.4 (C14), 53.8 (C9), 83.1 (C17), 124.1 (C4), 171.0 (C5), 174.4 (*C*=O, glycine), 199.6 (*C*=O, C3) ppm [2]. ESI–MS $[M+H]^+$ m/z: 346.2383; calculated $[M+H]^+$ m/z: 346.2377.

Polymer P1: yield 212.0 mg (56%). ATR–FTIR (solid) υ max: 3270 (N–H), 2882 (C–H), 1768 (C=O), 1457 (C–H bend), 1108 (C–O–C), 1043 (P=N) cm^{-1}; ^1H NMR (300 MHz, 298 K, $CDCl_3$): δ = 0.83 (t, 12H, 4′C*H*$_3$- + 8′C*H*$_3$- + 12′C*H*$_3$-), 1.10 (br, 14H, CH_3– of PPO groups of Jeffamine M-1000), 3.35 (s, 6H, CH_3O– end groups of Jeffamine M-1000), 3.62 (m, 139H,

polyalkylene oxide –CH_2–), 7.58 (d, 0.63H, ($C_6\boldsymbol{H}_5$)$_3$P=N– end group) ppm; ^{13}C NMR (75 MHz, $CDCl_3$): δ = 11.4 (5CH_3-), 11.9 (8CH_3-), 12.4 (7CH_3-), 17.4 (C+), 19.8 (4′CH_3- + 8′CH_3-), 20.9 (C4), 21.2 (C2′), 22.7 (12′CH_3-), 22.8 (12′CH_3-), 23.9 (2CH_3-), 24.6 (C6′), 25.0 (C10′), 28.1 (C12′), 32.9 (C4′ + C8′), 34.1 (C3), 37.4-37.5 (C3′ + C5′ + C7′ + C9′), 39.5 (C1′ + C11′), 46.2 (–NH–CH2–COO–), 59.1 (–O–CH_2–CH(CH_3)– of Jeffamine M1000), 70.7 (–O–CH_2–CH_2–O– of Jeffamine M1000), 72.0 (–O–CH_2–CH(CH_3)– of Jeffamine M1000), 75.2 (C2), 117.6 (C9), 118.7 (C5), 121.3 (C7), 122.7 (C8), 144.7 (C9), 145.6 (C10), 149.7 (C6) [39]; ^{31}P NMR (121 MHz, $CDCl_3$): δ = 0.0 ppm. GPC* (g/mol) M_n = 14174, M_w = 19121. Glass transition temperature (T_g) = -15.0 °C, melting temperature (T_m) = 26.7 °C.

Polymer P2: yield 200 mg (45%). ATR–FTIR (solid) υ max: 3265 (N–H), 2883 (C–H), 1768 (C=O), 1465 (C–H bend), 1108 (C–O–C), 1044 (P=N) cm^{-1}; ^{1}H NMR (300 MHz, $CDCl_3$): δ = 0.82 (t, 4H, 4′CH_3- + 8′CH_3- + 12′CH_3-), 1.10 (br, 14H, CH_3– of PPO groups of Jeffamine M-1000), 3.35 (s, 6H, CH_3O– end groups of Jeffamine M-1000), 3.61 (m, 136H, polyalkylene oxide –CH_2–), 7.59 (d, 0.76H, ($C_6\boldsymbol{H}_5$)$_3$P=N– end group) ppm; ^{31}P NMR (121 MHz, $CDCl_3$): δ = 0.84 ppm. GPC* (g/mol) M_n = 18775, M_w = 24068. T_g = -14.6 °C, T_m = 28.4 °C.

Polymer P3: yield 130 mg (48%). ATR–FTIR (solid) υ max: 3289 (N–H), 2866 (C–H), 1739 (C=O), 1672 (C=O, ketone C3 of testosterone), 1454 (C–H bend), 1107 (C–O–C), 1041 (P=N) cm^{-1}; ^{1}H NMR (300 MHz, $CDCl_3$): δ = 0.81 (s, 1H, H18), 1.11 (br, 14H, CH_3– of PPO groups of Jeffamine M-1000), 3.36 (s, 6H, CH_3O– end groups of Jeffamine M-1000), 3.62 (m, 139H, polyalkylene oxide –CH_2–), 7.59 (d, 0.73H, ($C_6\boldsymbol{H}_5$)$_3$P=N– end group) ppm; ^{31}P NMR (121 MHz, $CDCl_3$): δ = 0.80 ppm. GPC* (g/mol) M_n = 17117, M_w = 25382. T_g = -14.5 °C, T_m = 26.8 °C.

* The molecular weights of polymers P1–P3 determined by GPC measurements calibrated against linear polystyrene standards deviated from the values calculated for the polymers, due to the different hydrodynamic volume of branched polyphosphazenes and linear polystyrene standards (20).

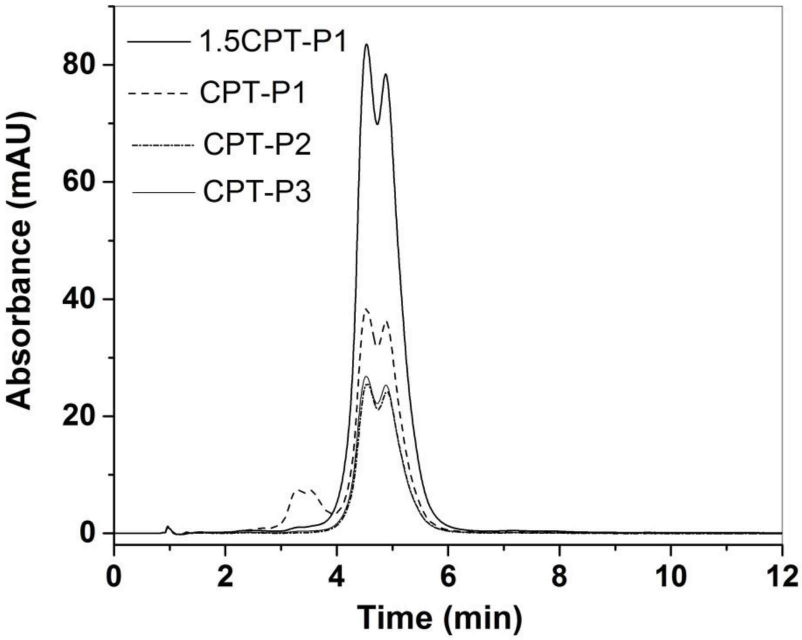

Figure S1. HPLC chromatograms at 370 nm of 1.5CPT-P1 at 0.0367 mg/mL, CPT-P1 at 0.064 mg/mL, CPT-P2 at 0.017 mg/mL, CPT-P3 at 0.017 mg/mL in PBS (see structures in Figure 1 of manuscript).

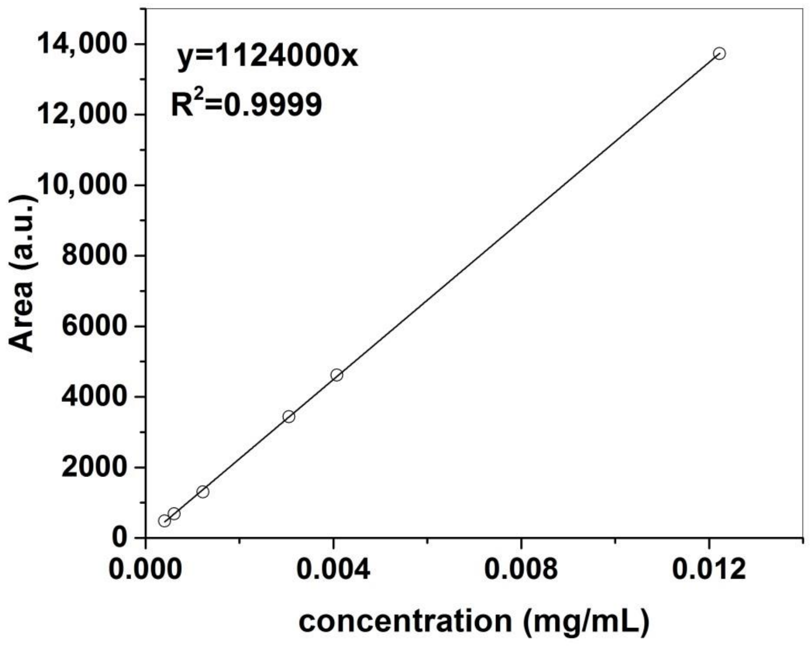

Figure S2. Calibration curve of CPT in PBS (pH 7.4) obtained from HPLC chromatograms at 254 nm.

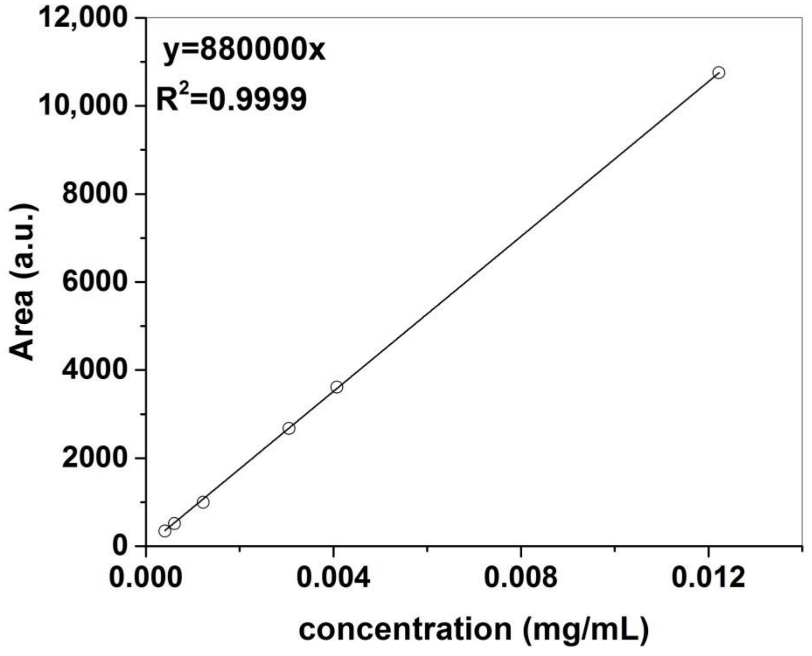

Figure S3. Calibration curve of CPT in PBS (pH 7.4) obtained from HPLC chromatograms at 370 nm.

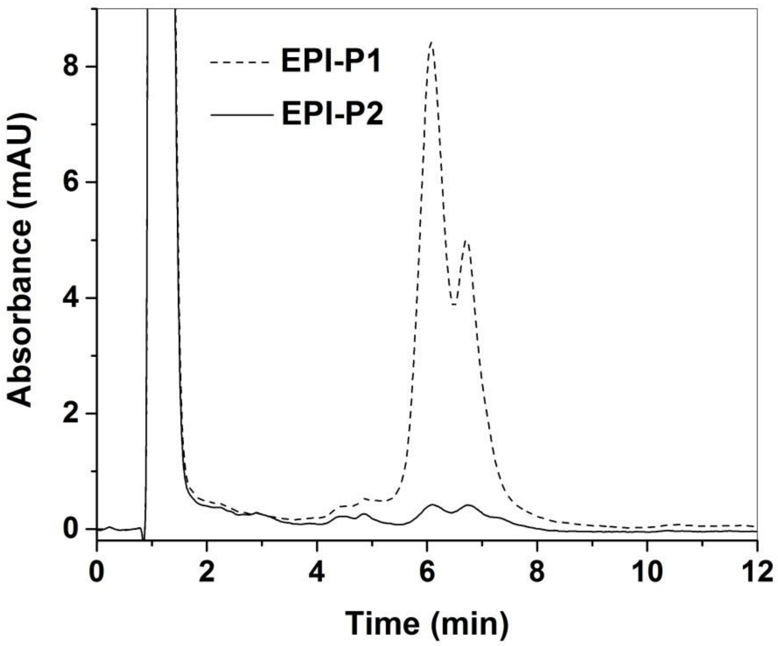

Figure S4. HPLC chromatograms at 254 nm of EPI-P1 at 0.053 mg/mL and EPI/P2 at 0.017 mg/mL in PBS (see structures in Figure 1 in manuscript).

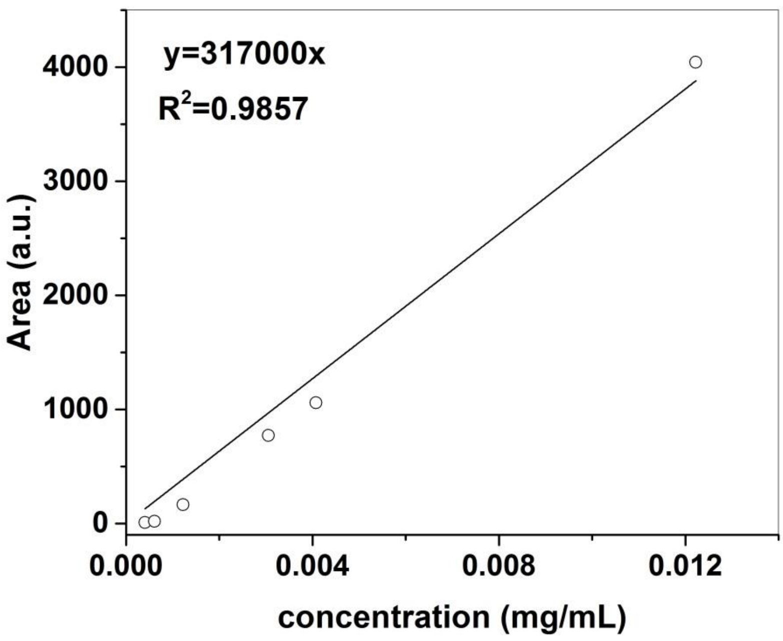

Figure S5. Calibration curve of EPI in PBS (pH 7.4) obtained from HPLC chromatograms at 254 nm.

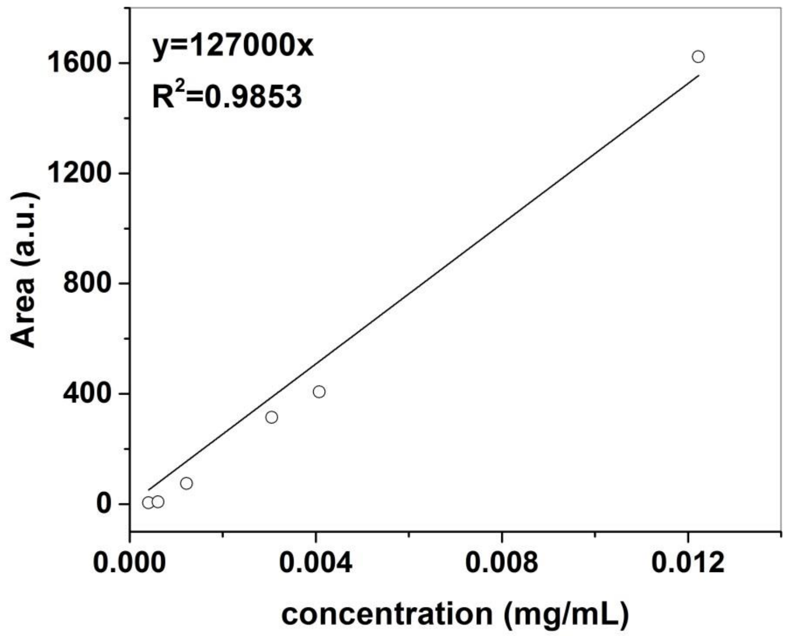

Figure S6. Calibration curve of EPI in PBS (pH 7.4) obtained from HPLC chromatograms at 475 nm.

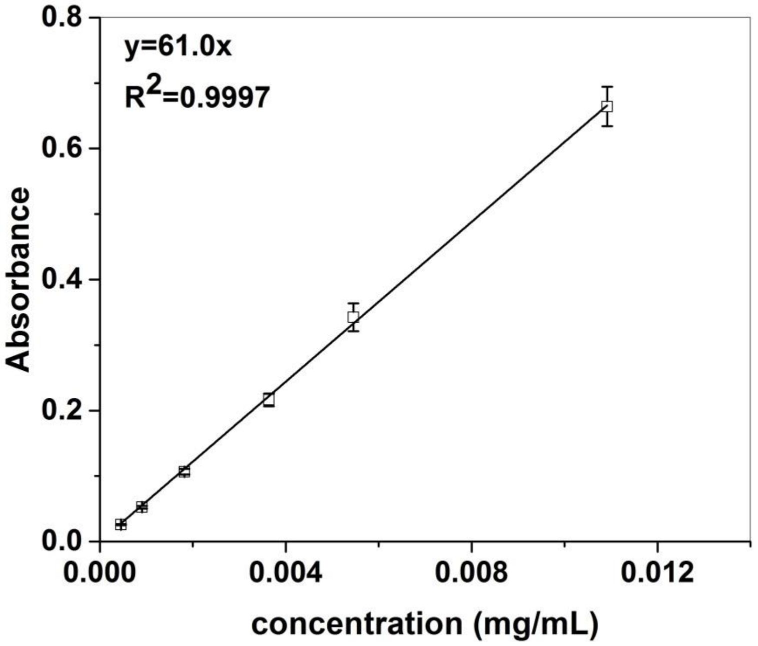

Figure S7. Calibration curve of testosterone in PBS (pH 7.4) (testosterone ε_{249}^{PBS}= 17594 M^{-1} cm^{-1}) (see structures in Figure 1 in manuscript).

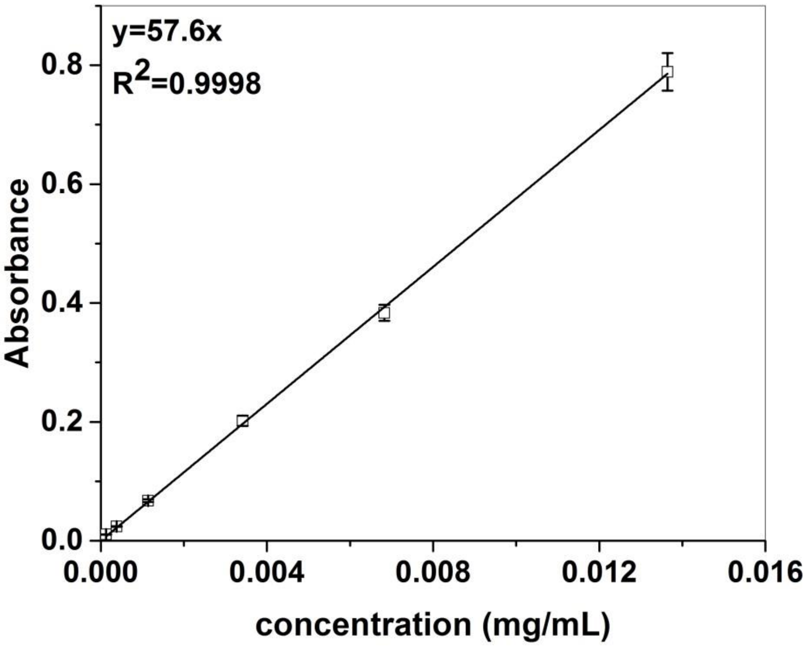

Figure S8. Calibration curve of testosterone in PBS (pH 6.0) (testosterone ε_{249}^{PBS}= 16613 M^{-1} cm^{-1}) (see structures in Figure 1 in manuscript).

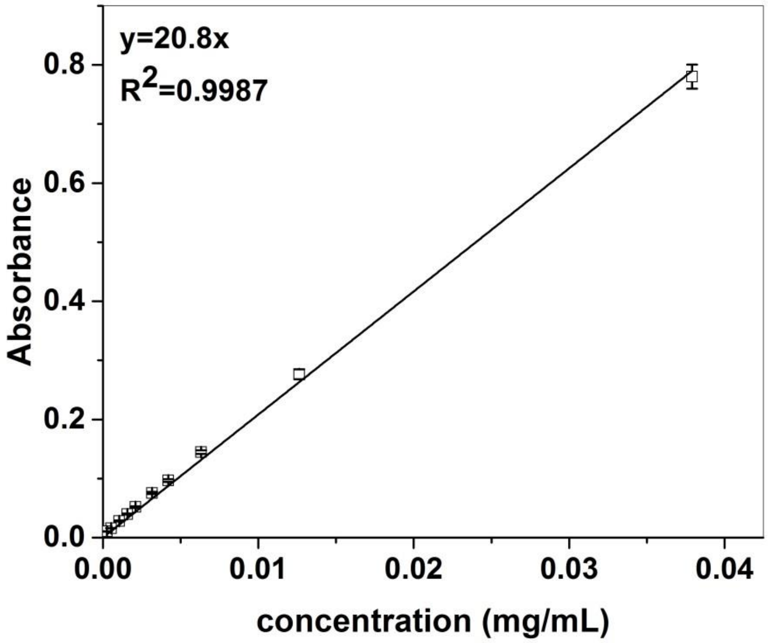

Figure S9. Calibration curve of DL-α-tocopherol in PBS (pH 7.4) (tocopherol ε_{290}^{PBS}= 8960 M^{-1} cm^{-1}) (see structures in Figure 1 in manuscript).

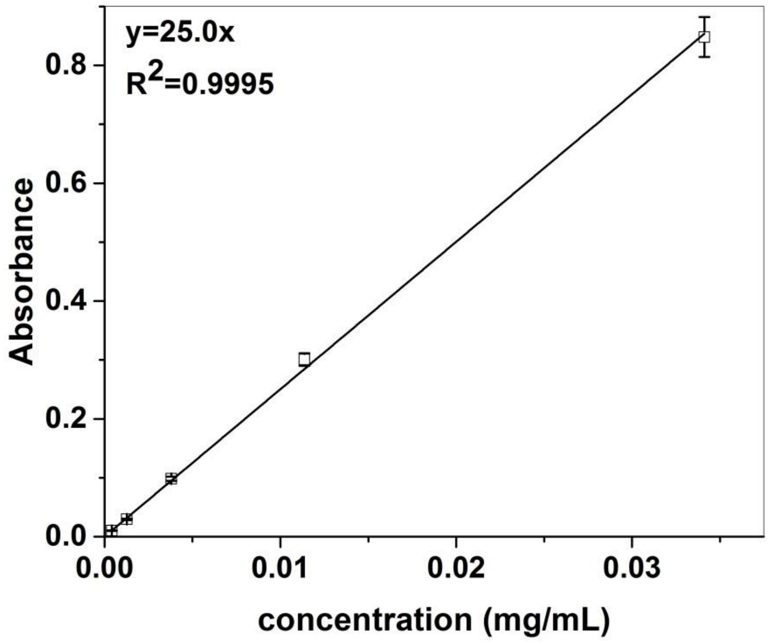

Figure S10. Calibration curve of DL-α-tocopherol in PBS (pH 6.0) (tocopherol ε_{290}^{PBS}= 10768 M^{-1} cm^{-1}) (see structures in Figure 1 in manuscript).

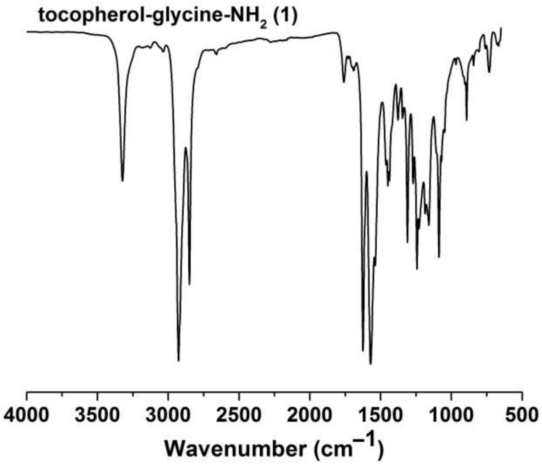

Figure S11. FTIR spectra of tocopherol-glycine-NH_2 (1) (see structures in Figure 1 in manuscript).

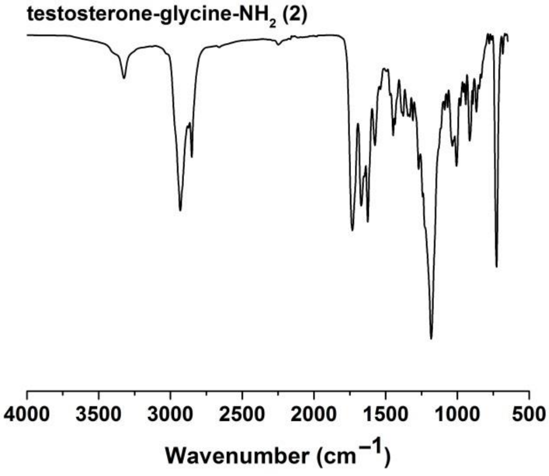

Figure S12. FTIR spectra of testosterone-glycine-NH_2 (2) (see structures in Figure 1 in manuscript).

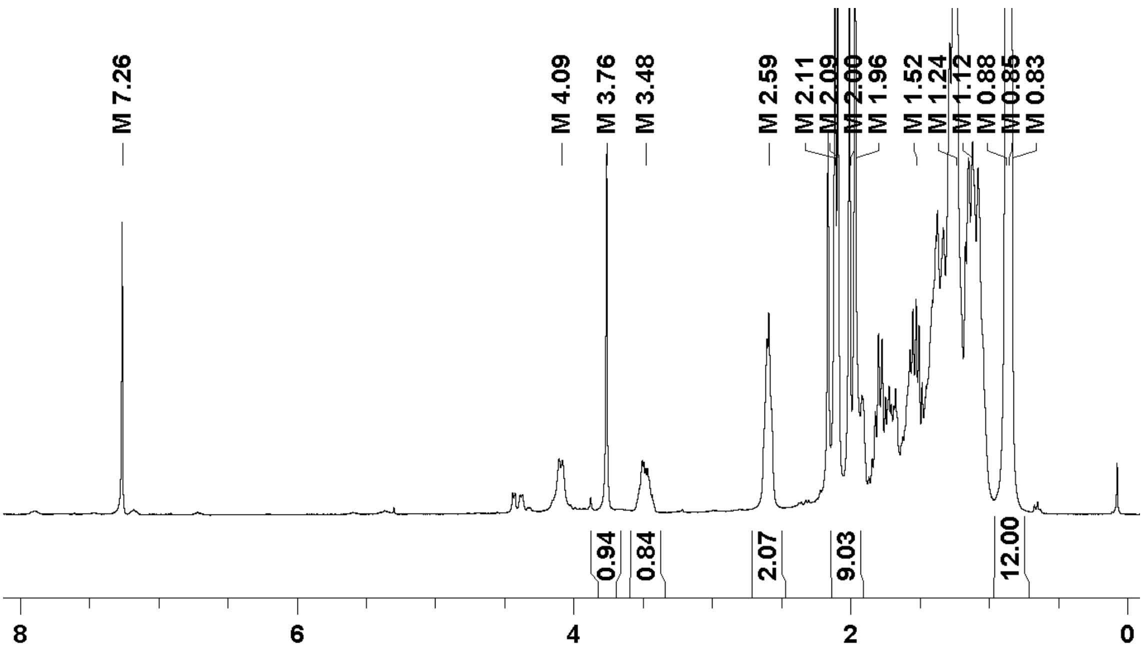

Figure S13. ^{1}H NMR (300 MHz, 298 K, $CDCl_3$) spectrum of tocopherol-glycine-NH_2 (1) (see structures in Figure 1 in manuscript).

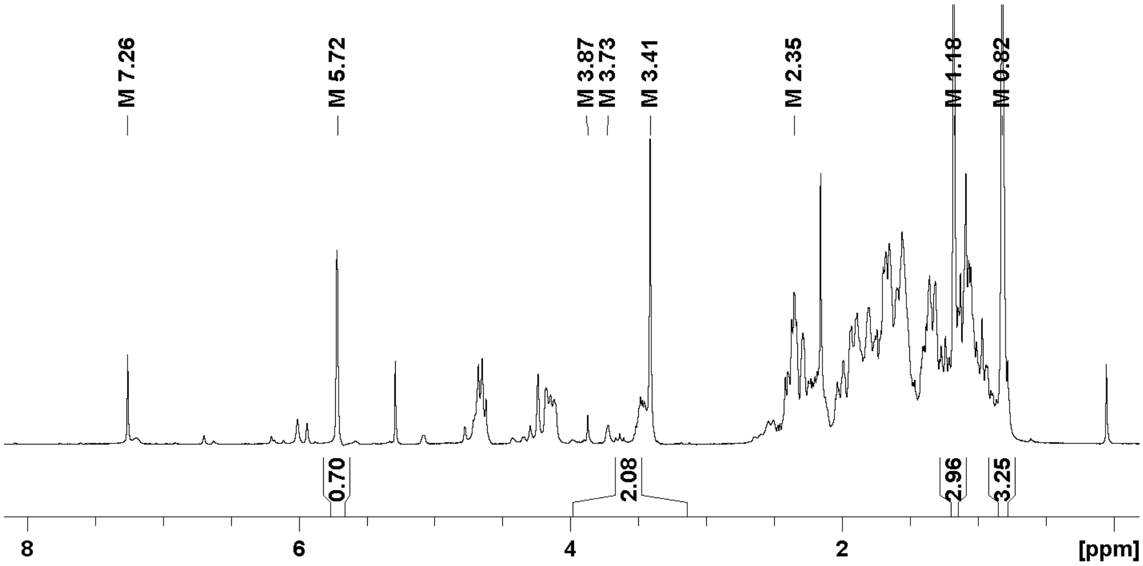

Figure S14. ^{1}H NMR (300 MHz, 298 K, $CDCl_3$) spectrum of testosterone-glycine-NH_2 (2) (see structures in Figure 1 in manuscript).

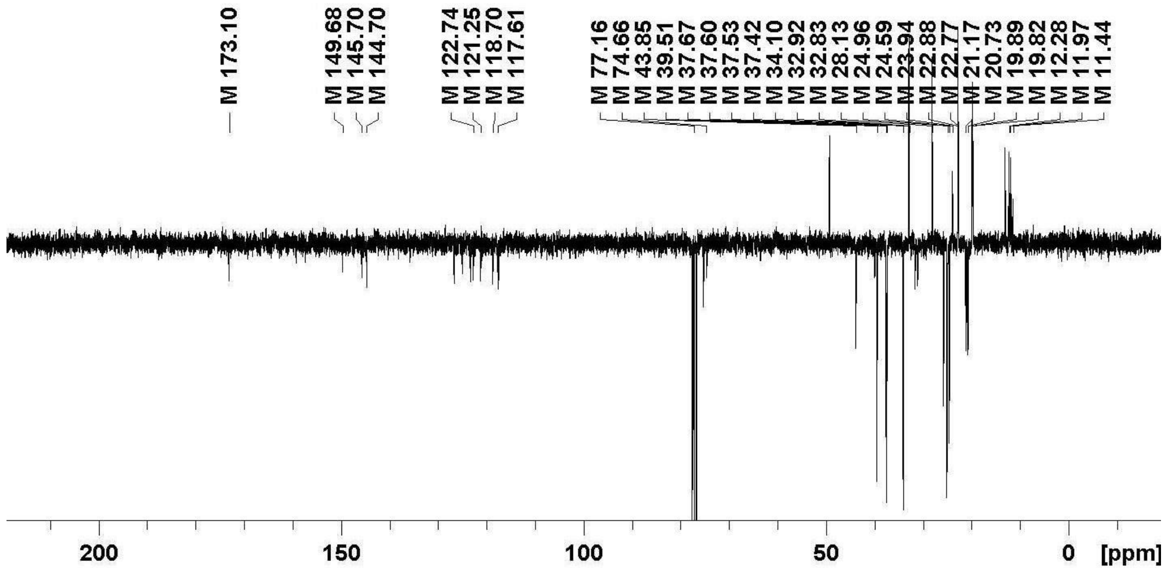

Figure S15. APT ^{13}C NMR (75 MHz, 298 K, $CDCl_3$) spectrum of tocopherol-glycine-NH_2 (1) (see structures in Figure 1 in manuscript).

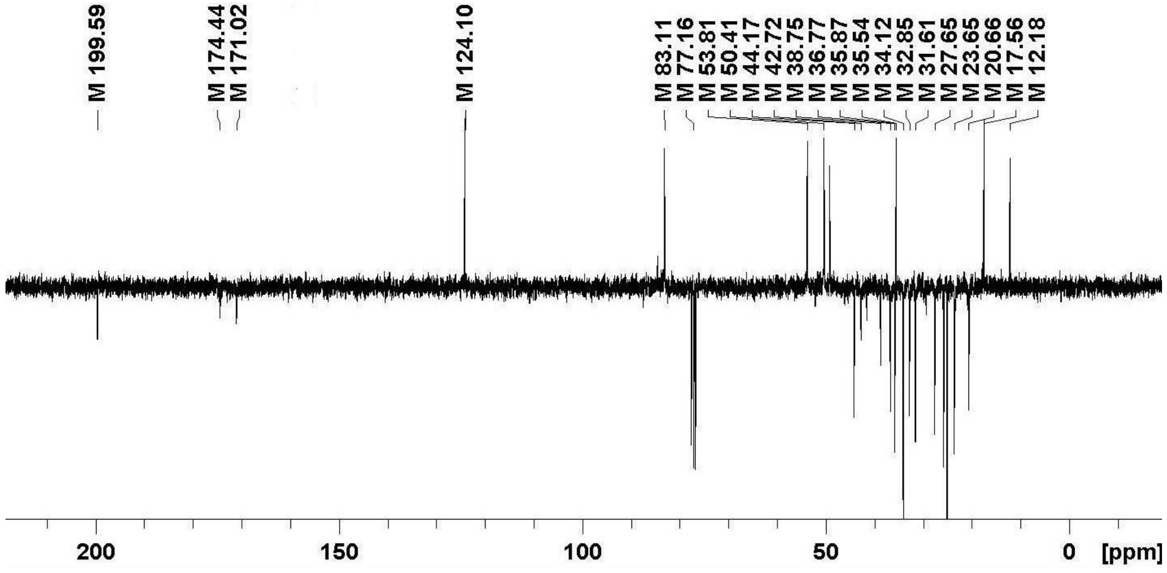

Figure S16. APT ^{13}C NMR (75 MHz, 298 K, $CDCl_3$) spectrum of testosterone-glycine-NH_2 (2) (see structures in Figure 1 in manuscript).

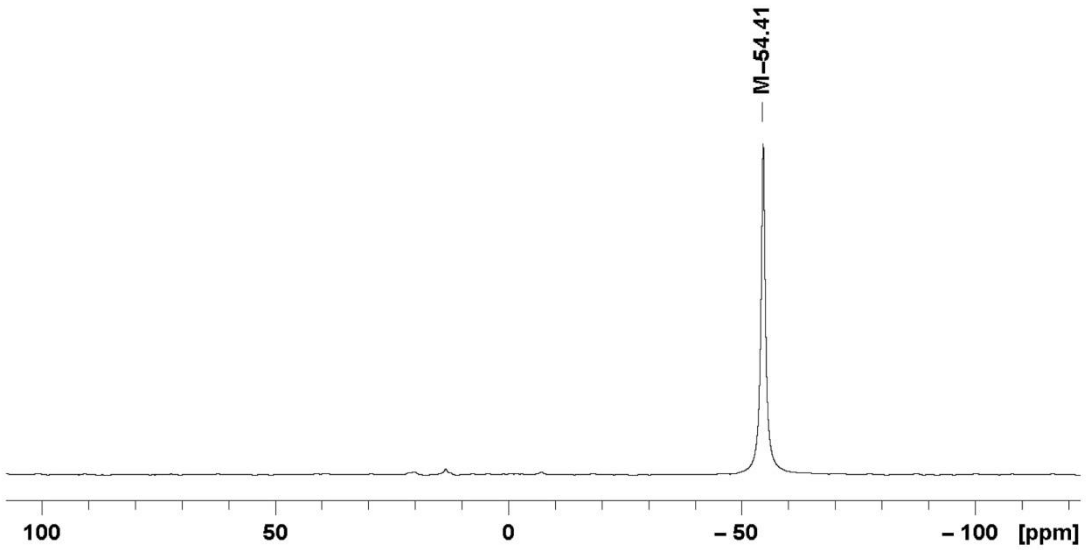

Figure S17. $^{31}P\{^1H\}$ NMR (121 MHz, 298 K, $CDCl_3$) spectrum of monomer trichlorophosphoranimine ($Cl_3P{=}N{-}Si(CH_3)_3$).

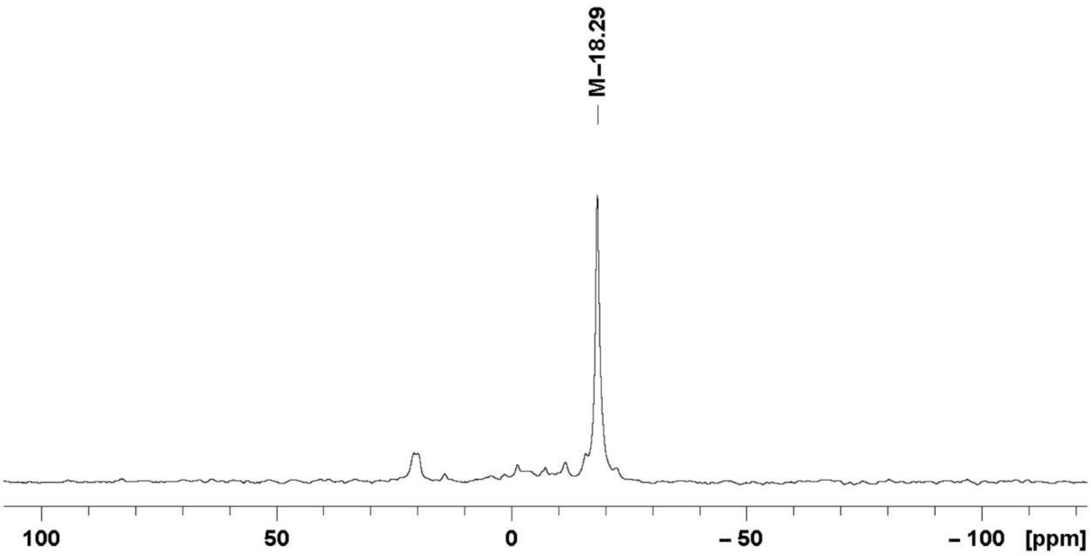

Figure S18. $^{31}P\{^1H\}$ NMR (121 MHz, 298 K, $CDCl_3$) spectrum of polydichlorophosphazene.

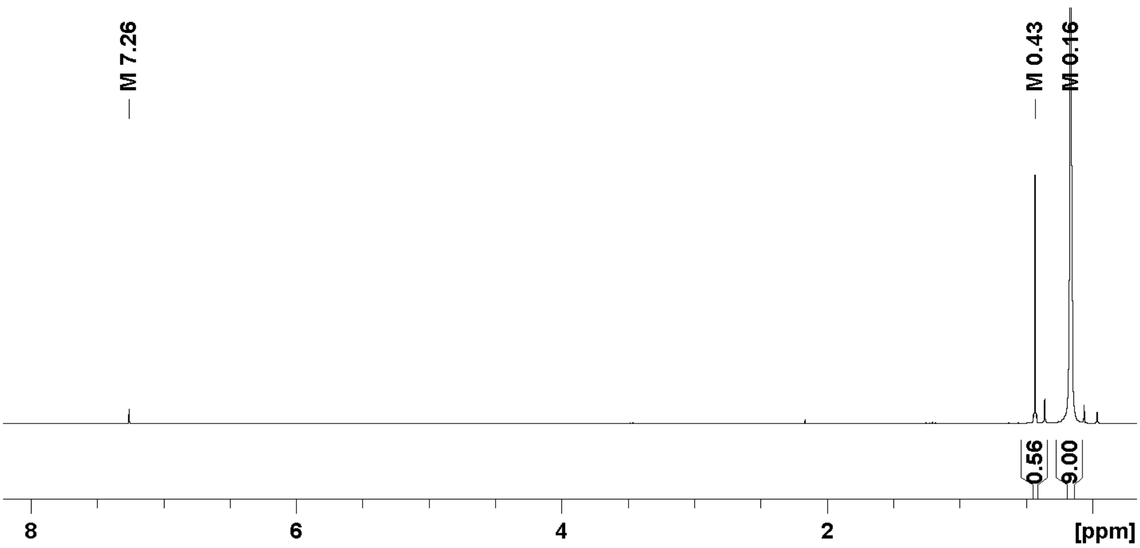

Figure S19. ^1H NMR (300 MHz, 298 K, $CDCl_3$) spectrum of monomer trichlorophosphoranimine ($Cl_3P{=}N{-}Si(CH_3)_3$).

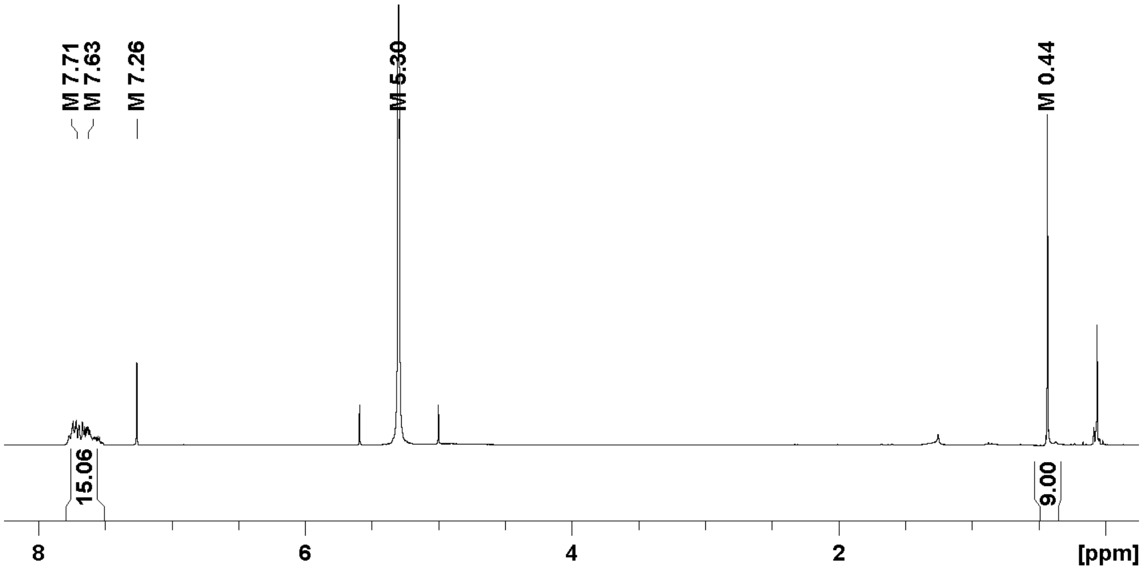

Figure S20. ¹H NMR (300 MHz, 298 K, $CDCl_3$) spectrum of polydichlorophosphazene.

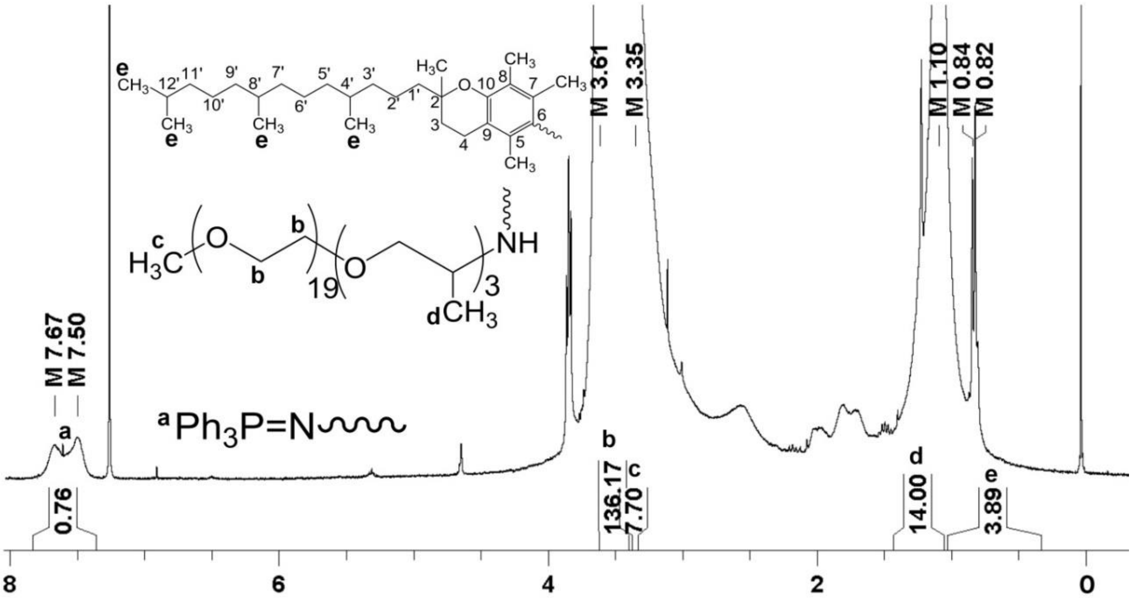

Figure S21. ¹H NMR (300 MHz, 298 K, $CDCl_3$) spectrum of polymer P2. DP was calculated as $DP = \frac{15}{I_{Ph_3P-}} = \frac{15}{Ia}$ (see structures in Figure 1 in manuscript).

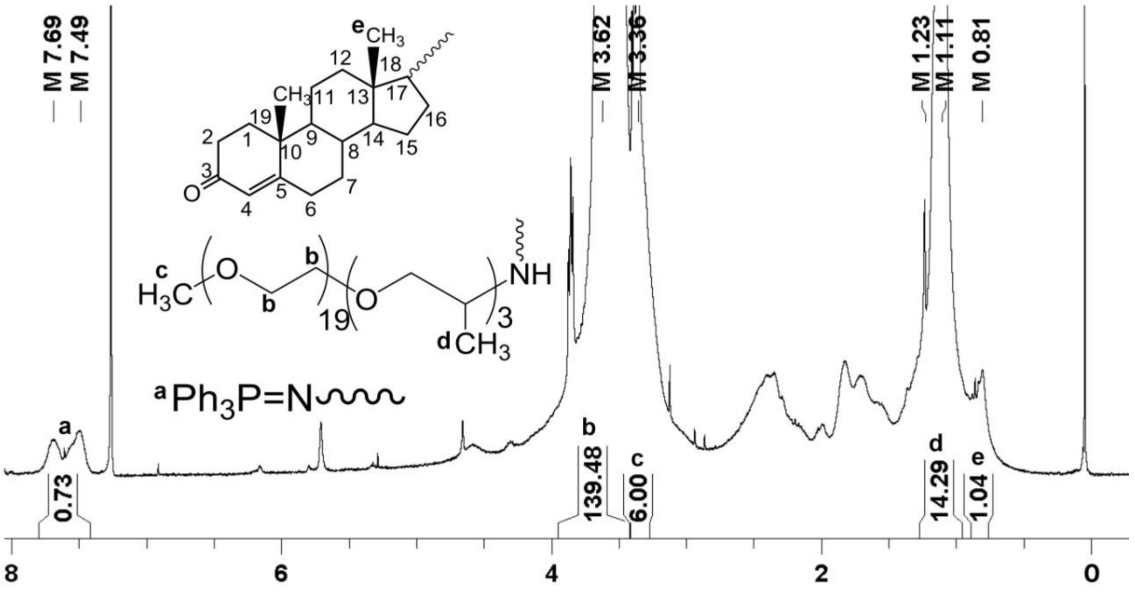

Figure S22. ^{1}H NMR (300 MHz, 298 K, $CDCl_3$) spectrum of polymer P3. DP was calculated as $DP = \frac{15}{I_{Ph_3P-}} = \frac{15}{Ia}$ (see structures in Figure 1 in manuscript).

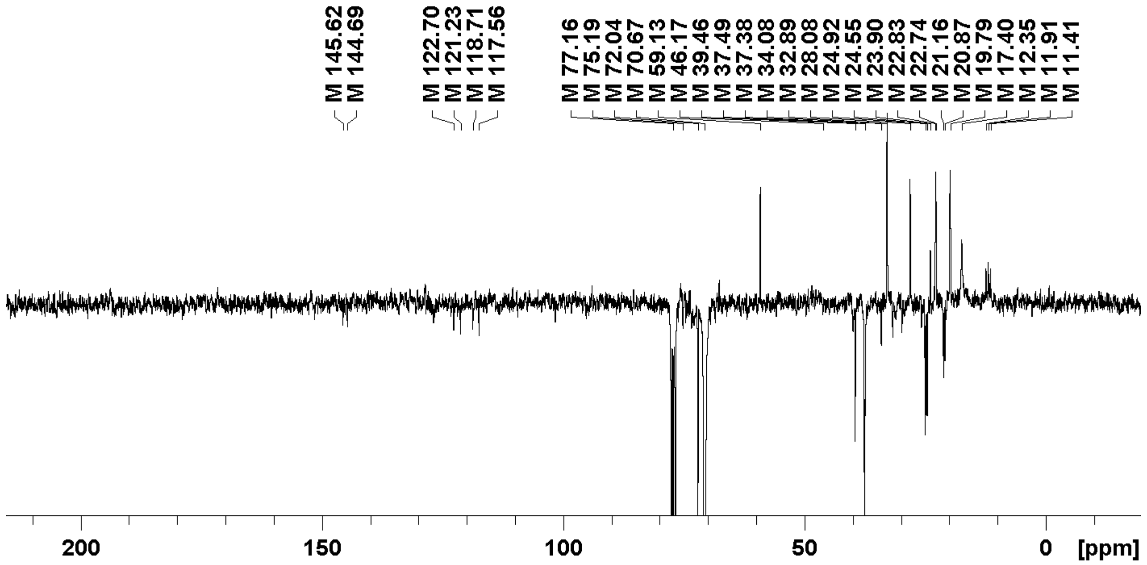

Figure S23. APT ^{13}C NMR (75 MHz, 298 K, $CDCl_3$) spectrum of polymer P1 (see structures in Figure 1 in manuscript).

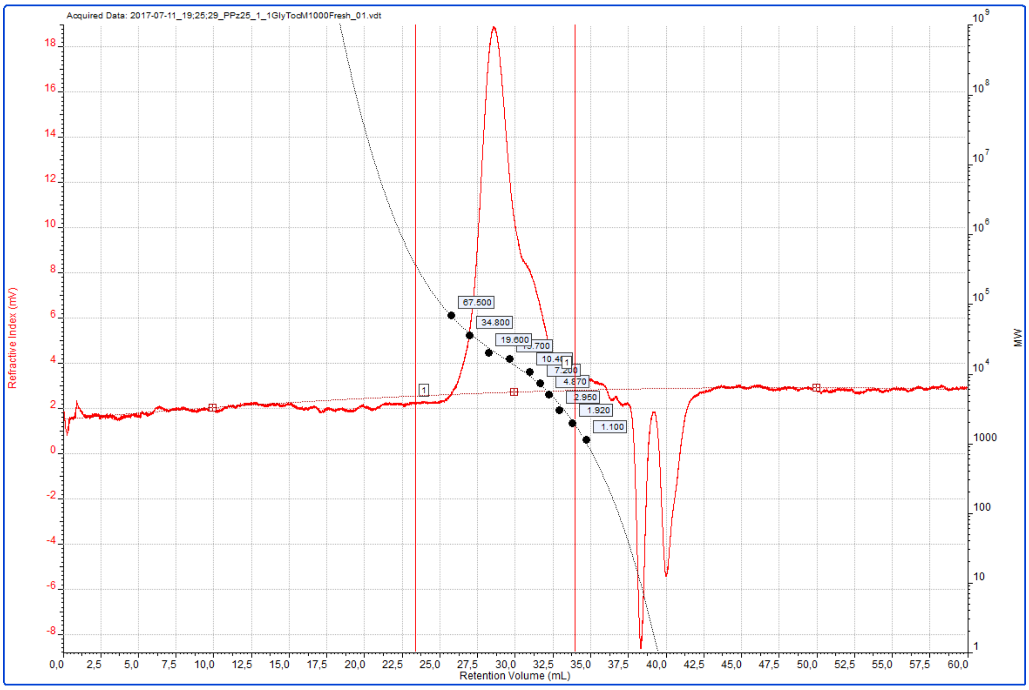

Figure S24. GPC chromatogram of polymer P1 (see structures in Figure 1 in manuscript).

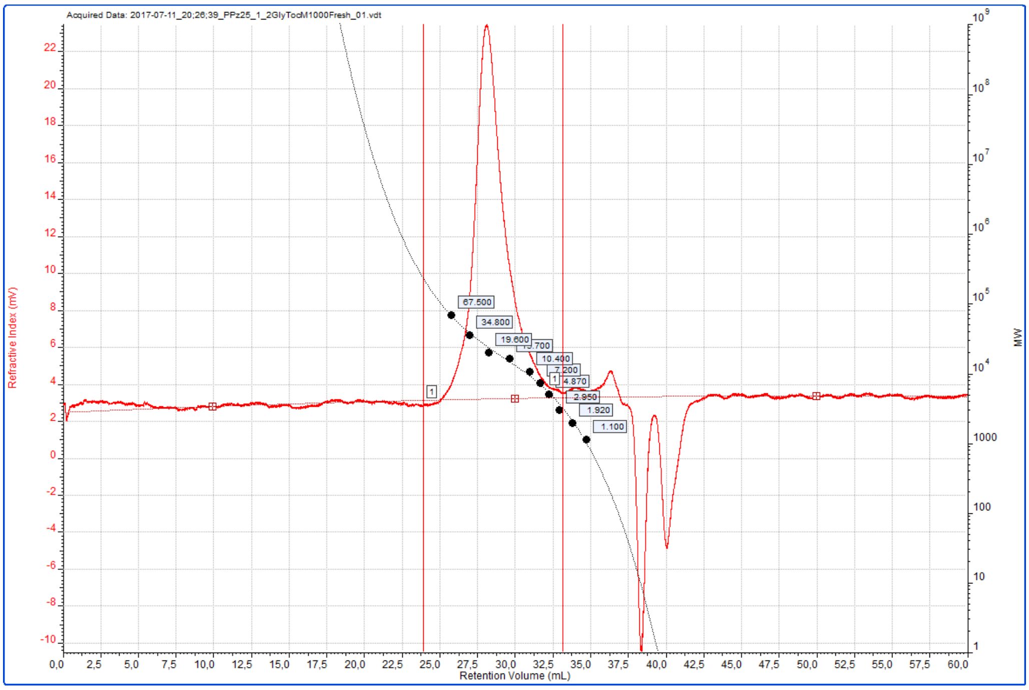

Figure S25. GPC chromatogram of polymer P2 (see structures in Figure 1 in manuscript).

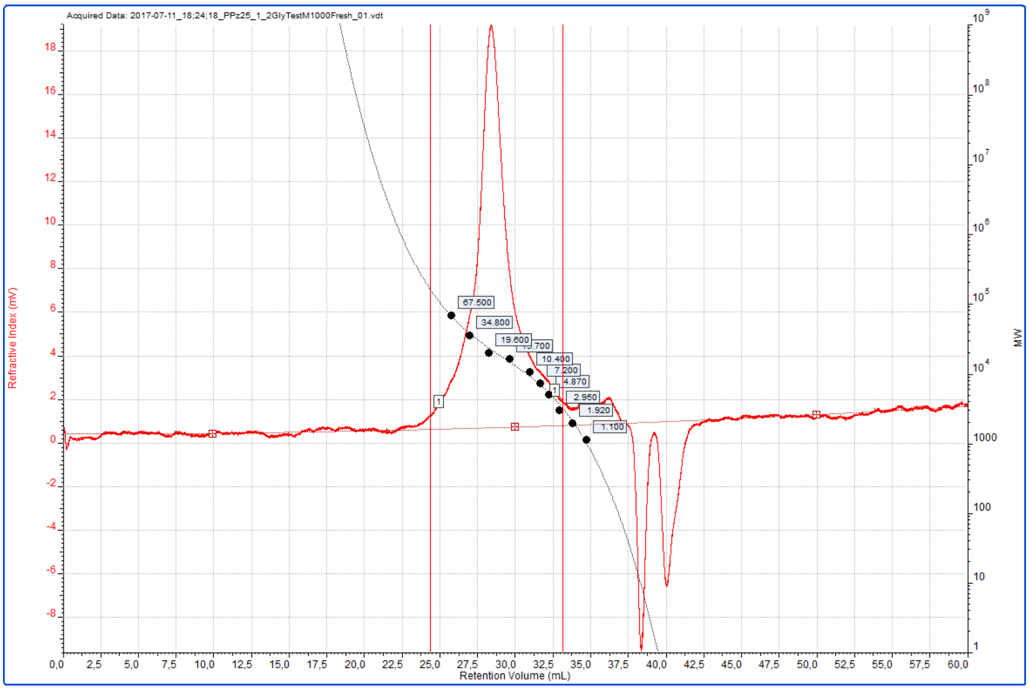

Figure S26. GPC chromatogram of polymer P3 (see structures in Figure 1 in manuscript).

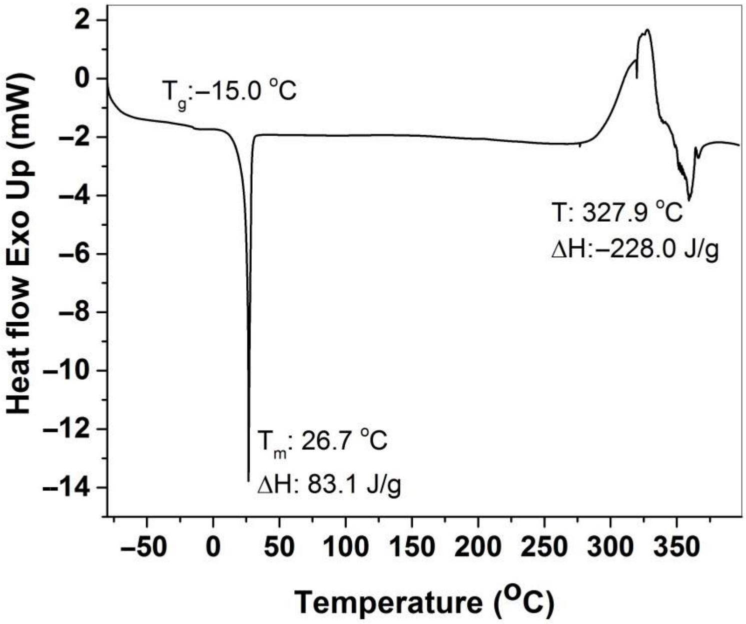

Figure S27. Calorimetry differential scanning curve of polymer P1 (see structures in Figure 1 in manuscript).

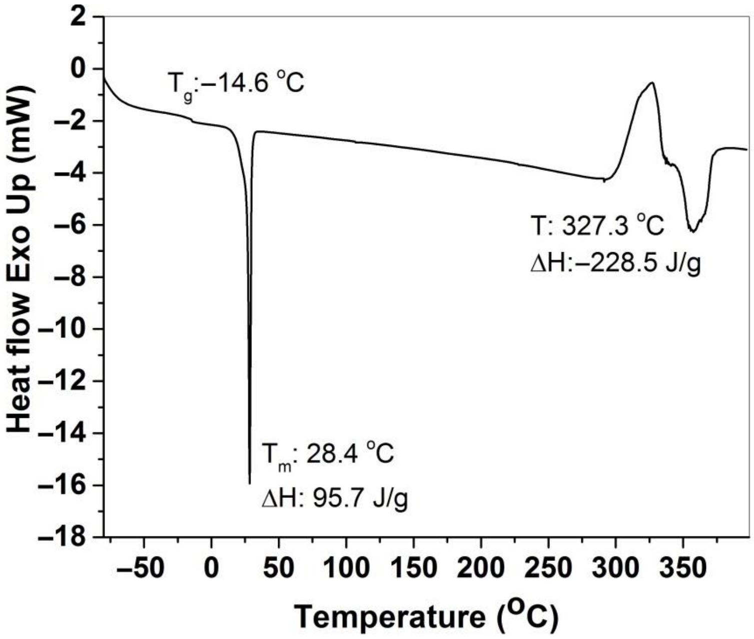

Figure S28. Calorimetry differential scanning curve of polymer P2 (see structures in Figure 1 in manuscript).

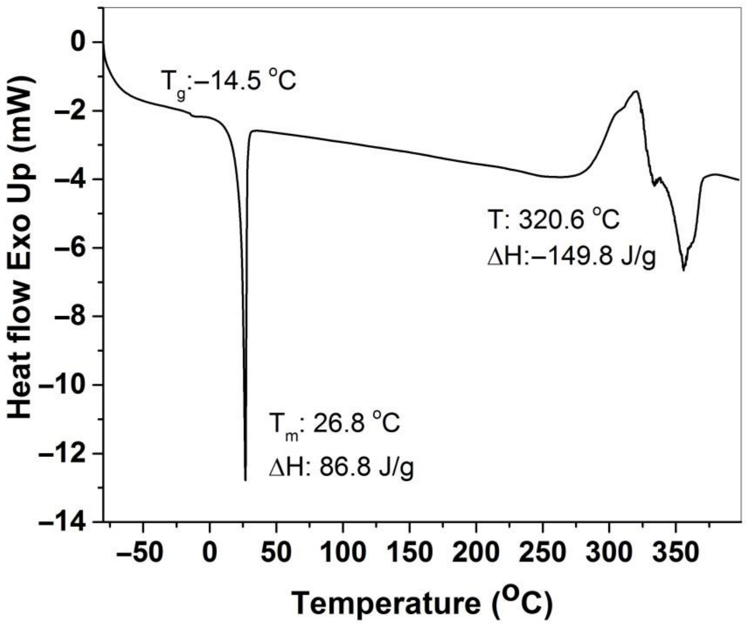

Figure S29. Calorimetry differential scanning curve of polymer P3 (see structures in Figure 1 in manuscript).

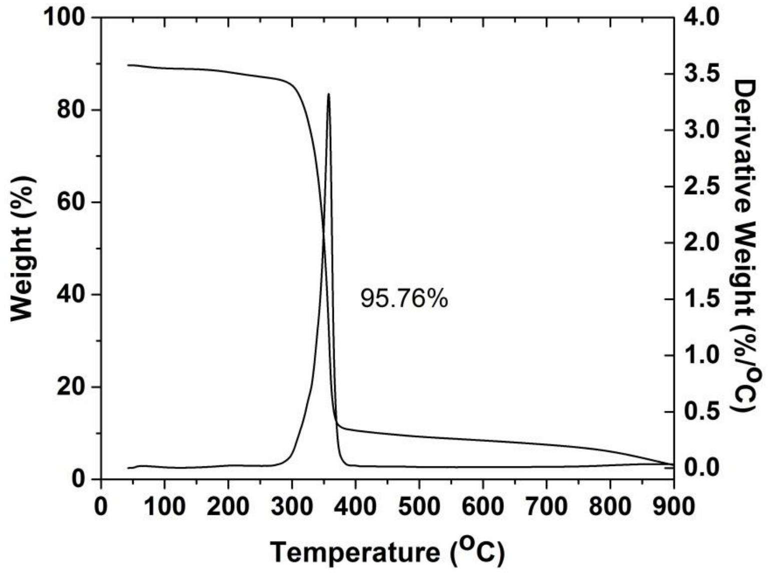

Figure S30. TGA curve of P1 (see structures in Figure 1 in manuscript).

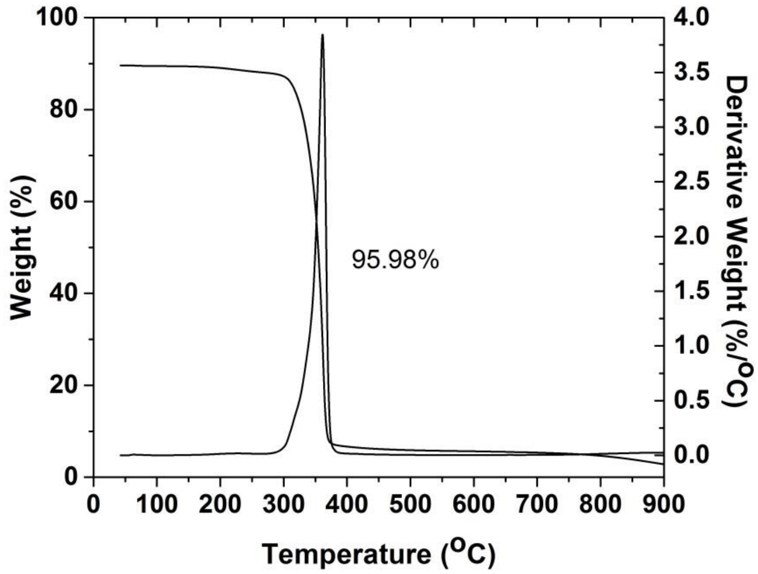

Figure S31. TGA curve of P2 (see structures in Figure 1 in manuscript).

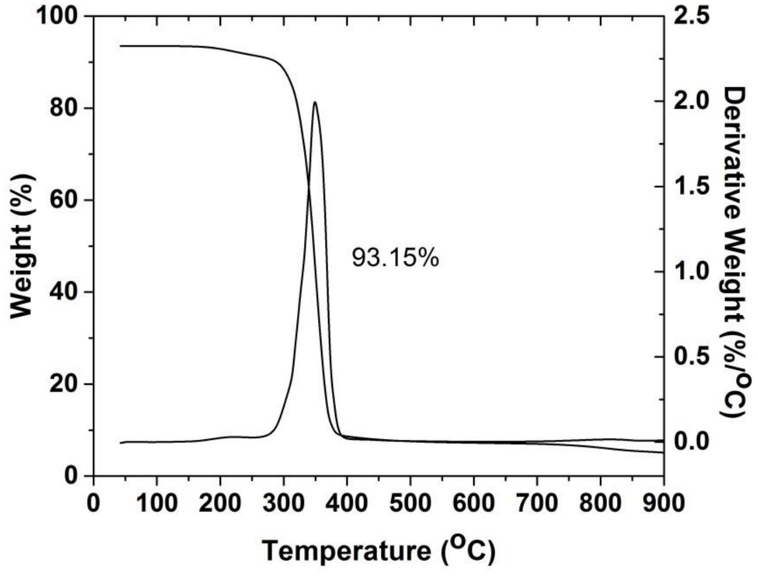

Figure S32. TGA curve of P3 (see structures in Figure 1 in manuscript).

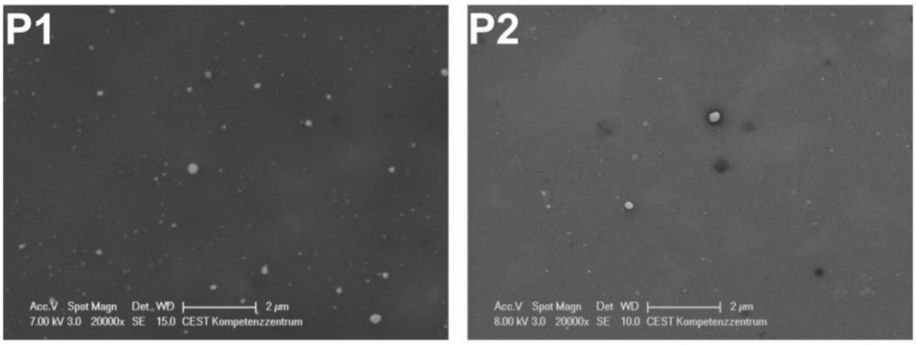

Figure S33. SEM micrographs of P1 and P2 dried nanoaggregates at 20,000× magnification (see structures in Figure 1 in manuscript).

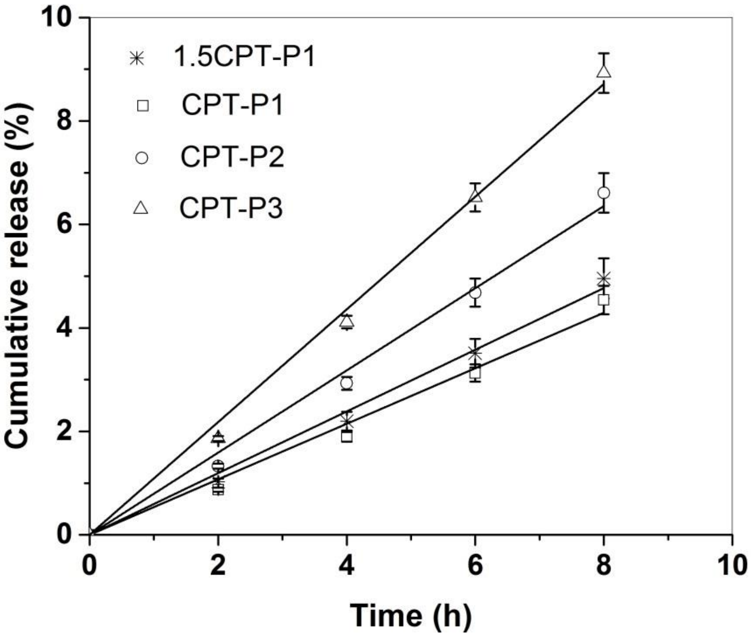

Figure S34. Linear fitting of in vitro CPT release profiles of CPT-loaded polyphosphazene nanoaggregates up to 8 h in PBS (pH 7.4) at 37 °C (see structures in Figure 1 in manuscript).

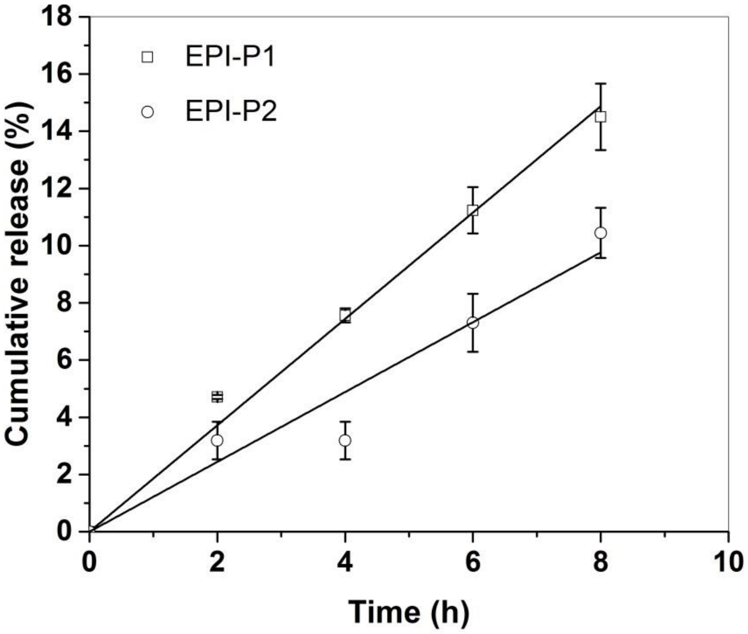

Figure S35. Linear fitting of in vitro EPI release profiles of EPI-loaded polyphosphazene nanoaggregates up to 8 h in PBS (pH 7.4) at 37 °C (see structures in Figure 1 in manuscript).

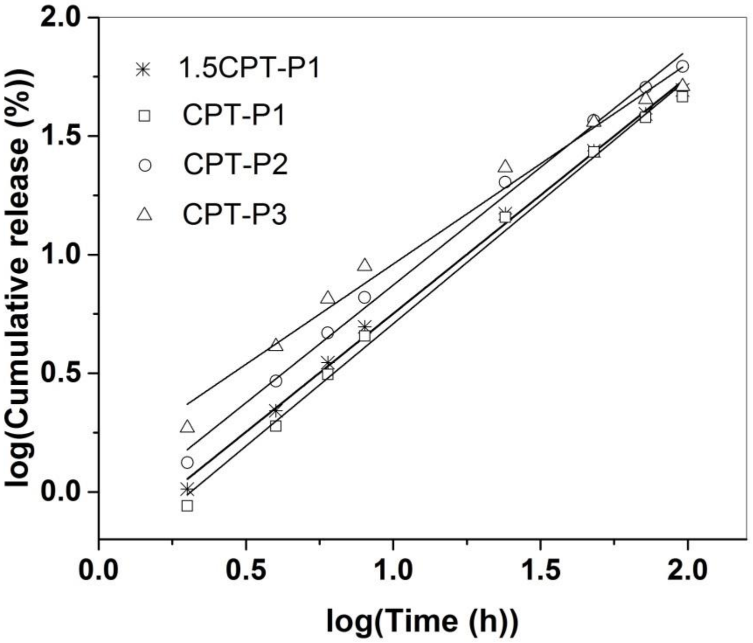

Figure S36. Linear fitting of log(cumulative release (%)) vs. log(time (hours)) of CPT-loaded polyphosphazene nanoaggregates up to 100 h in PBS (pH 7.4) at 37 °C (see structures in Figure 1 in manuscript).

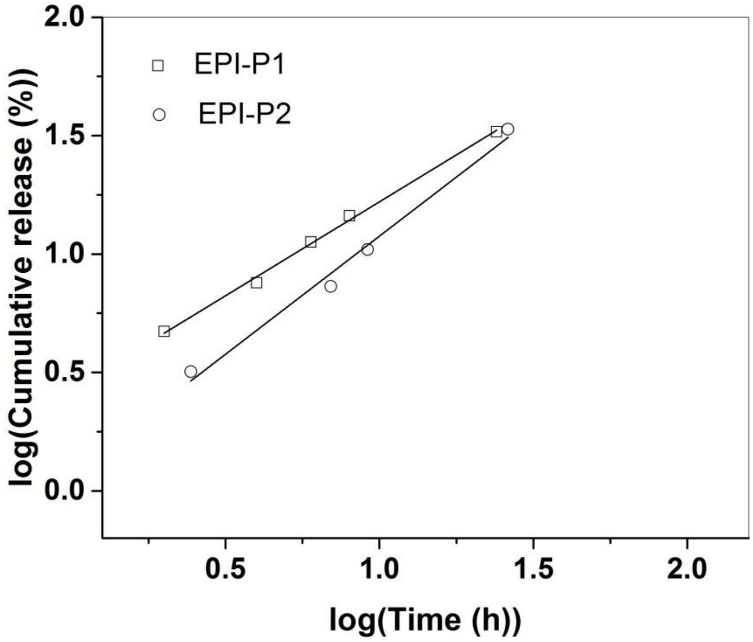

Figure S37. Linear fitting of log(cumulative release (%)) vs. log(time (hours)) of EPI-loaded polyphosphazene nanoaggregates up to 24 h in PBS (pH 7.4) at 37 °C (see structures in Figure 1 in manuscript).

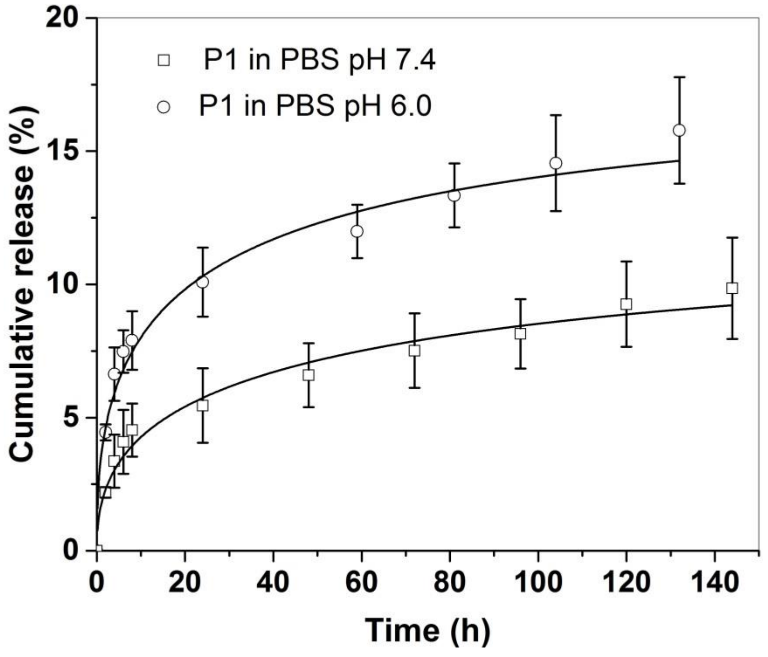

Figure S38. In vitro release profiles of tocopherol from P1 aggregates in PBS (pH 7.4 and 6.0) at 37 °C, adjusted to a SWeibull2 function (see structures in Figure 1 in manuscript).

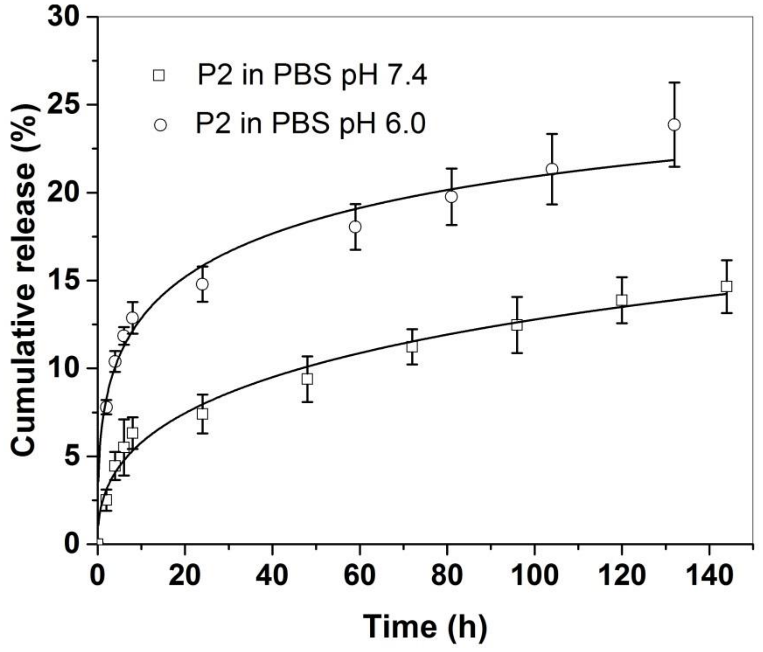

Figure S39. In vitro release profiles of tocopherol from P2 aggregates in PBS (pH 7.4 and 6.0) at 37 °C, adjusted to a SWeibull2 function (see structures in Figure 1 in manuscript).

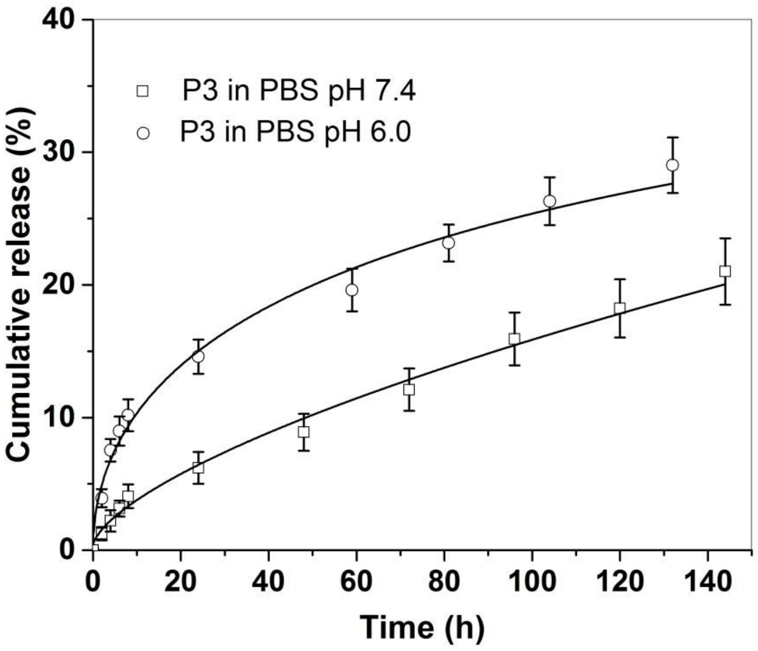

Figure S40. In vitro release profiles of testosterone from P3 aggregates in PBS (pH 7.4 and 6.0) at 37 °C, adjusted to a SWeibull2 function (see structures in Figure 1 in manuscript).

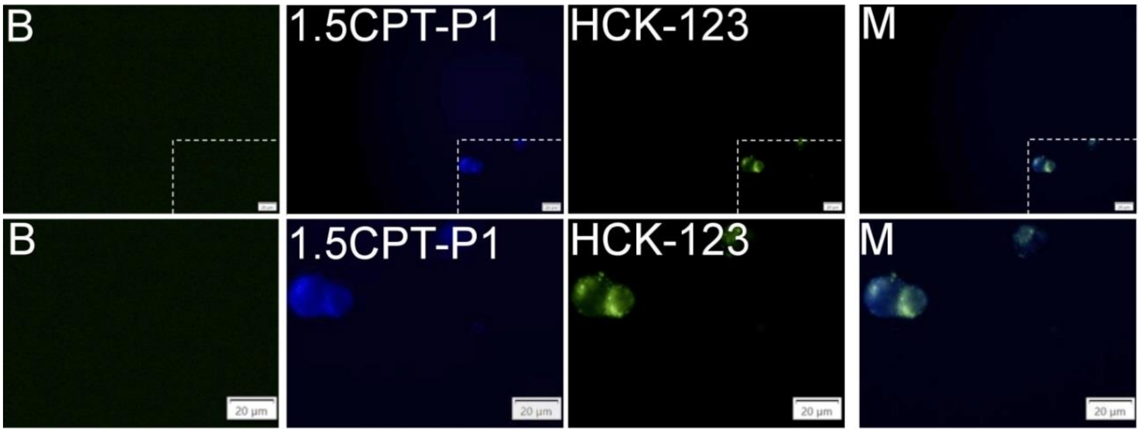

Figure S41. MCF-7 cells' fluorescence images and slices of cells without particles and LysoTracker (B). Cells with 0.1 mg/mL of 1.5CPT-P1 aggregates, 50 nM of LysoTracker Yellow HCK-123 and merged pictures (M). Scale bars represent 20 μm (see structures in Figure 1 in manuscript).

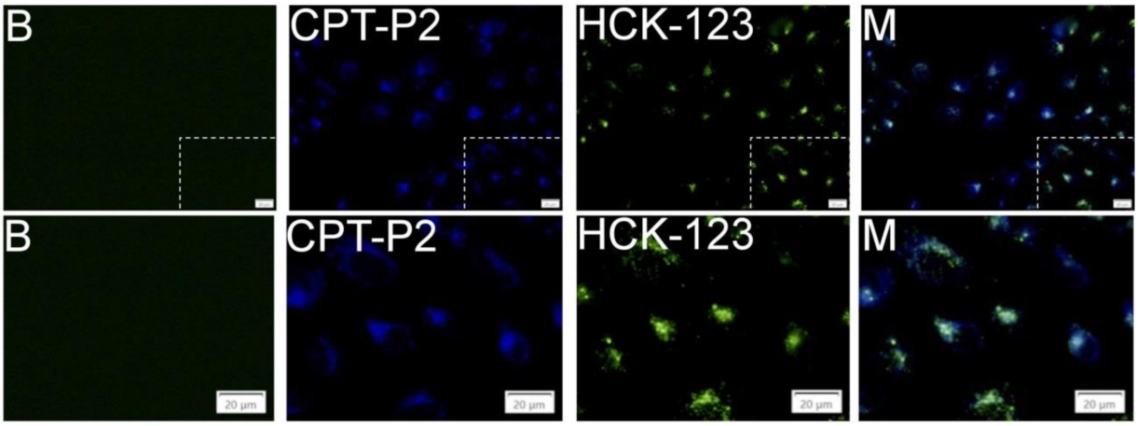

Figure S42. MCF-7 cells' fluorescence images and slices of cells without particles and LysoTracker (B). (a) Cells with 0.1 mg/mL of CPT-P2 aggregates, 50 nM of LysoTracker Yellow HCK-123 and merged pictures (M). Scale bars represent 20 μm (see structures in Figure 1 in manuscript).

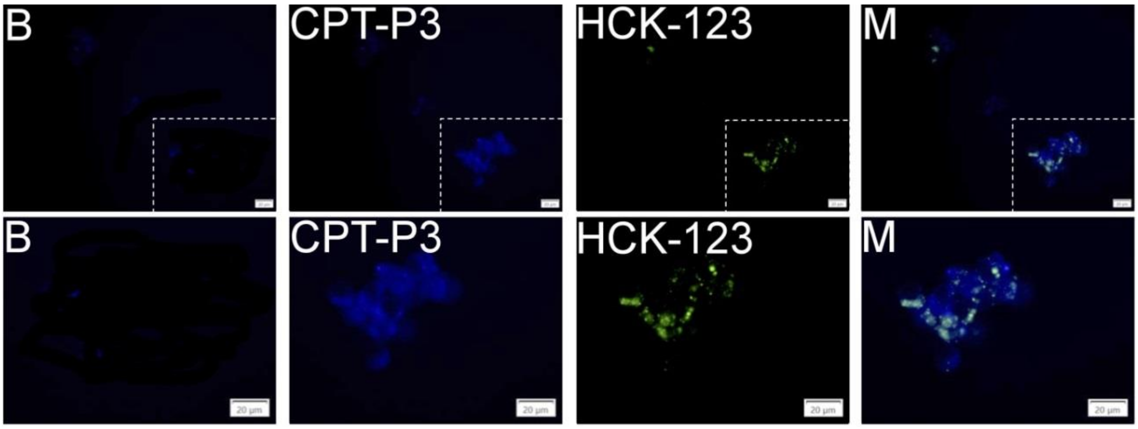

Figure S43. MCF-7 cells' fluorescence images and slices of cells without particles and LysoTracker (B). (a) Cells with 0.1 mg/mL of CPT-P3 aggregates, 50 nM of LysoTracker Yellow HCK-123 and merged pictures (M). Scale bars represent 20 μm (see structures in Figure 1 in manuscript).

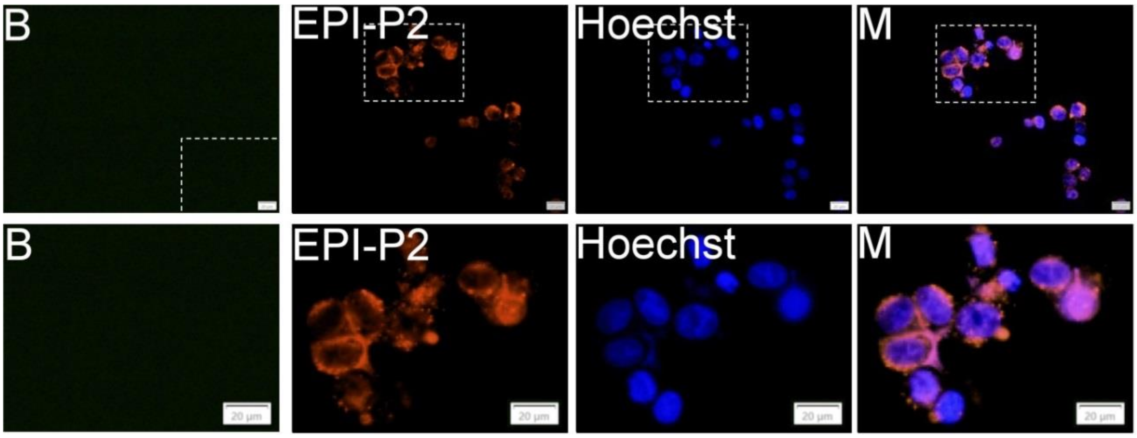

Figure S44. MCF-7 cells' fluorescence images and slices of cells without particles and Hoechst (B). Cells with 0.1 mg/mL of EPI-P2 aggregates, 1 μg/mL of Hoechst 33342 and merged pictures (M). Scale bars represent 20 μm (see structures in Figure 1 in manuscript).

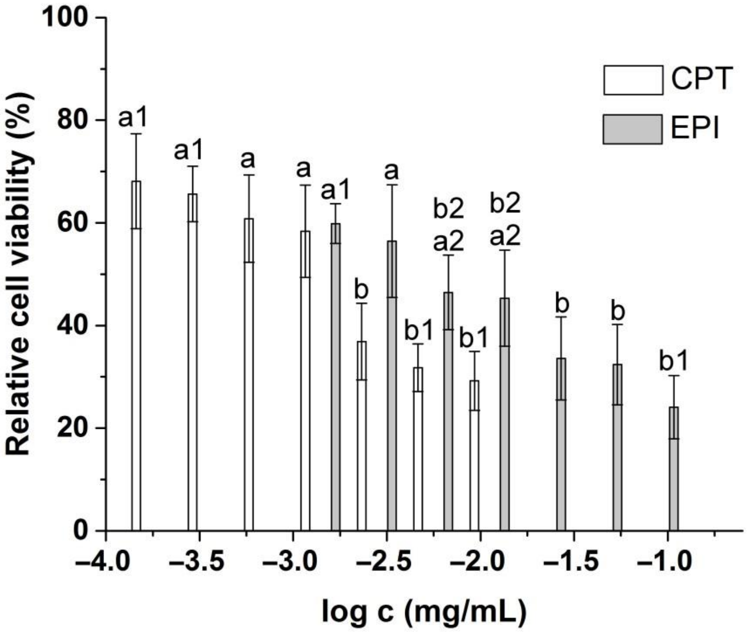

Figure S45. Relative cell viability of MCF-7 breast cancer cells treated with parent CPT or EPI. Data represent the mean ± standard deviation ($n = 3$). Means with no significant differences are presented with the same letter ($p > 0.05$). Means with significant differences are presented with different letters or the same letter but different numbers following the letter ($p < 0.05$).

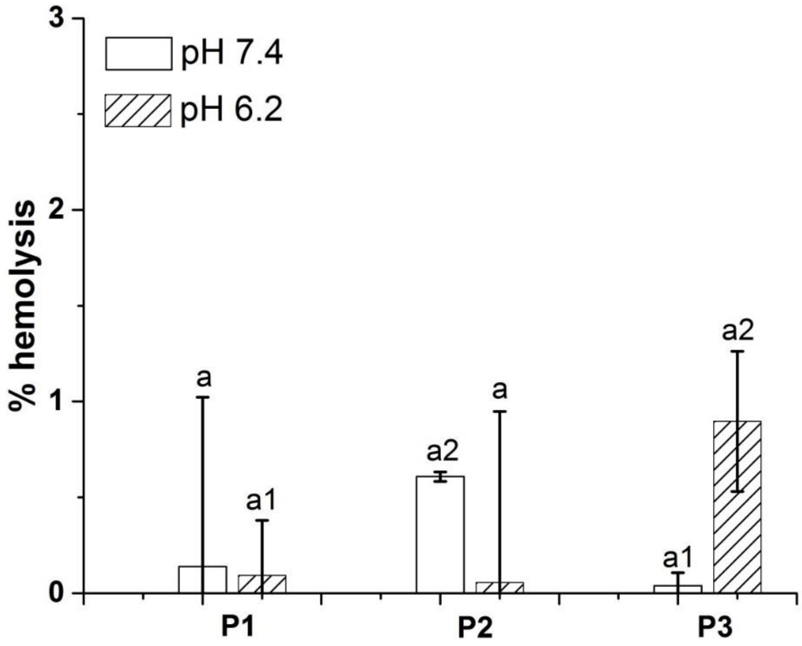

Figure S46. Percentage of hemolysis of RBC treated with 0.1 mg/mL of blank P1–P3 polyphosphazene nanoaggregates in PBS at pH 7.4 and 6.2 (see structures in Figure 1 in manuscript). Data represent the mean ± standard deviation (n = 3). Means with no significant differences are presented with the same letter ($p > 0.05$). Means with significant differences are presented with different letters or the same letter but different numbers following the letter ($p < 0.05$).

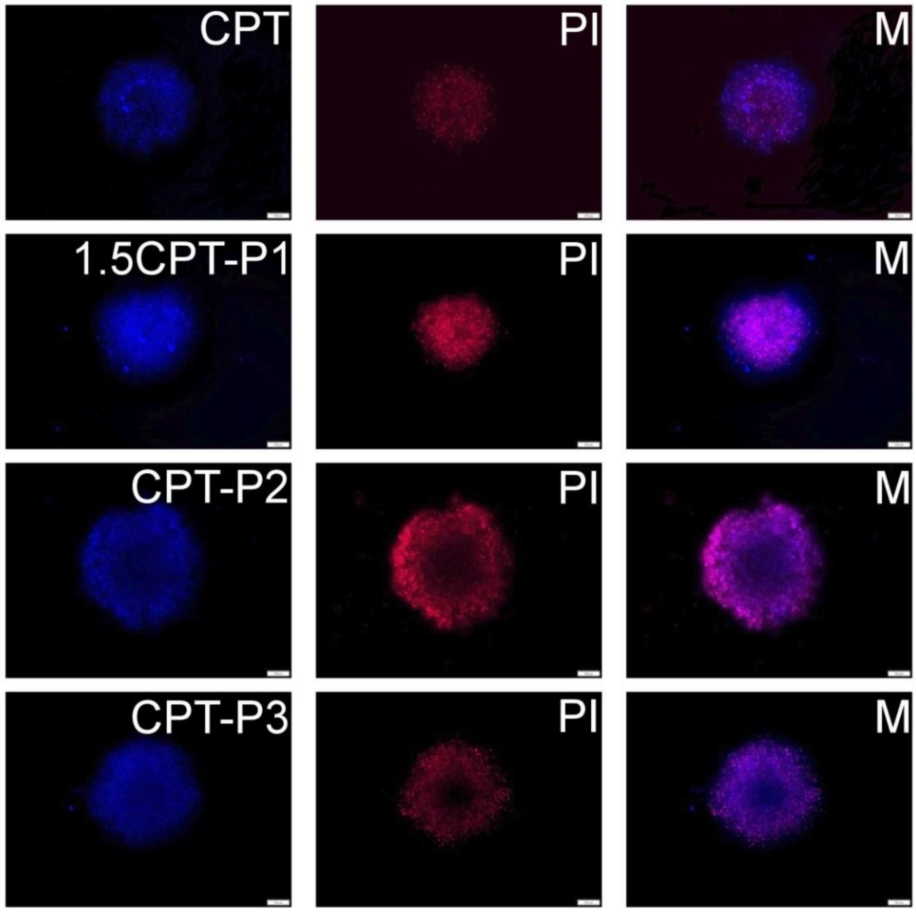

Figure S47. MCF-7 spheroid fluorescence images with 0.1 mg/mL of CPT or CPT-loaded aggregates, propidium iodide (PI) and merged pictures (M). Scale bars represent 100 μm (see structures in Figure 1 in manuscript).

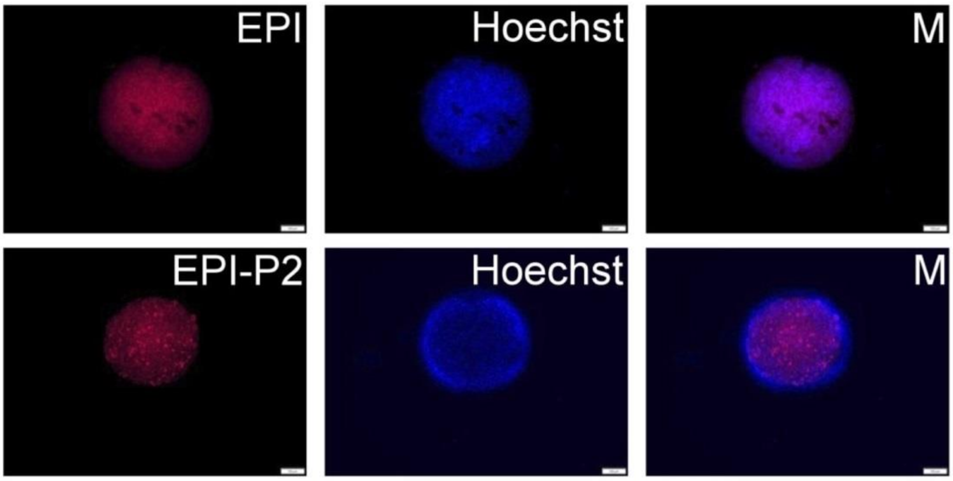

Figure S48. MCF-7 spheroid fluorescence images with 0.1 mg/mL of EPI or EPI-P2 aggregates, Hoechst 33342 and merged pictures (M). Scale bars represent 100 μm (see structures in Figure 1 in manuscript).

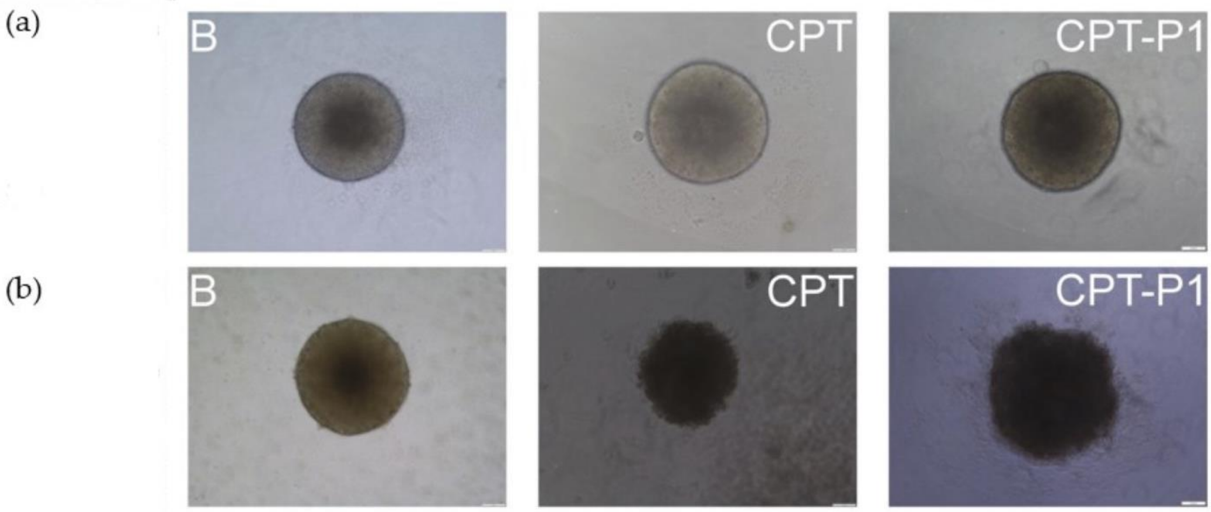

Figure S49. MCF-7 spheroids' bright field images without particles (B) or with 0.1 mg/mL of CPT or CPT-P1 nanoaggregates at 0 h (a) and 72 h (b). Scale bars represent 100 μm (see structures in Figure 1 in manuscript).

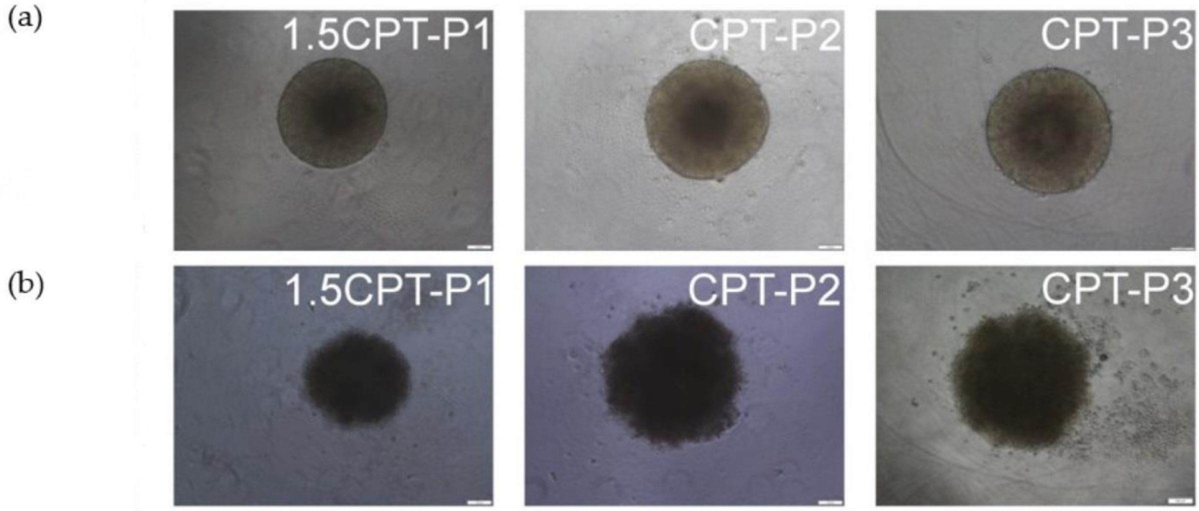

Figure S50. MCF-7 spheroids' bright field images with 0.1 mg/mL of CPT-loaded nanoaggregates at 0 h (a) and 72 h (b). Scale bars represent 100 μm (see structures in Figure 1 in manuscript).

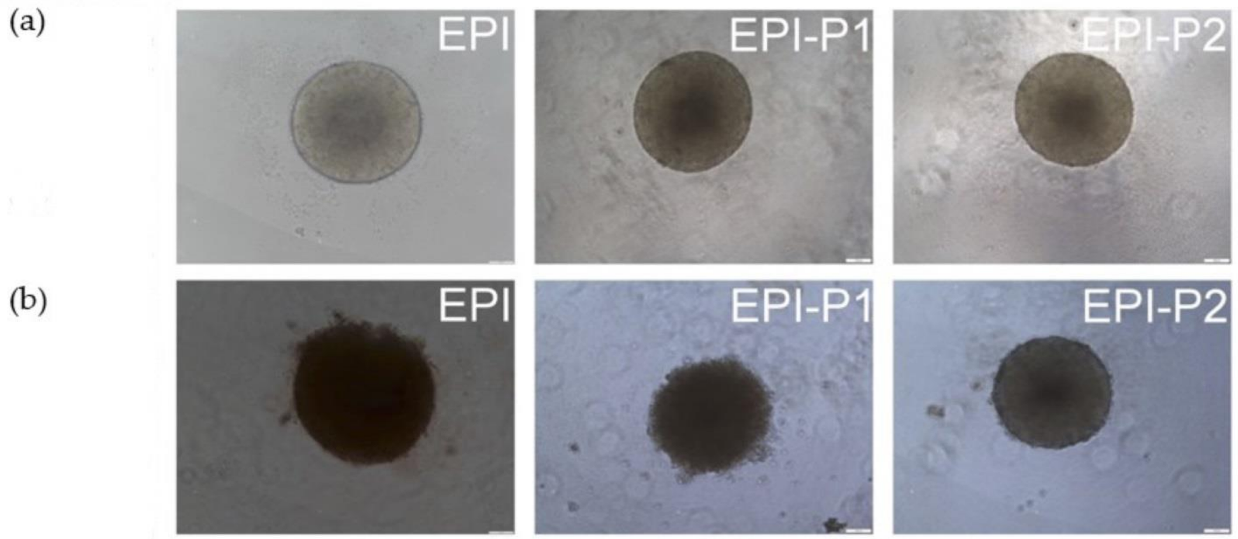

Figure S51. MCF-7 spheroids' bright field images with 0.1 mg/mL of EPI or EPI-loaded nanoaggregates. Scale bars represent 100 μm (see structures in Figure 1 in manuscript).

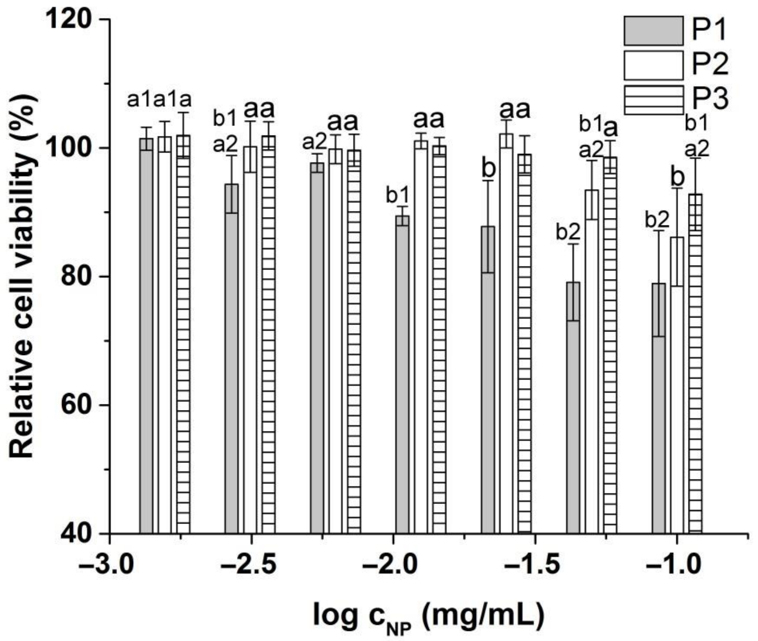

Figure S52. Relative cell viability of primary human lung fibroblasts treated with blank P1–P3 polyphosphazene nanoaggregates (see structures in Figure 1 in manuscript). Data represent the mean ± standard deviation (n = 3). Means with no significant differences are presented with the same letter ($p > 0.05$). Means with significant differences are presented with different letters or the same letter but different numbers following the letter ($p < 0.05$).

References

1. Lankhorst, P.P.; Netscher, T.; Duchateau, A.L.L. A Simple ^{13}C NMR method for the discrimination of complex mixtures of stereoisomers: All eight stereoisomers of α-tocopherol resolved. *Chirality* **2015**, *27*, 850–855, doi:10.1002/chir.22535.
2. Hayamizu, K.; Ishii, T.; Yanagisawa, M. Complete assignments of the ^{1}H and ^{13}C NMR spectra of testosterone and 17α-methyltestosterone and the ^{1}H parameters obtained from the 600 MHz spectra. *Magn. Reson. Chem.* **1990**, *28*, 250–256.
